# A data-driven approach for evaluating multi-modal therapy in traumatic brain injury

**DOI:** 10.1038/srep42474

**Published:** 2017-02-16

**Authors:** Jenny Haefeli, Adam R. Ferguson, Deborah Bingham, Adrienne Orr, Seok Joon Won, Tina I. Lam, Jian Shi, Sarah Hawley, Jialing Liu, Raymond A. Swanson, Stephen M. Massa

**Affiliations:** 1 Brain and Spinal Injury Center (BASIC), Department of Neurological Surgery, University of California, San Francisco, CA, United States; 2 San Francisco Veterans Affairs Medical Center, San Francisco, CA, United States; 3 Department of Neurology, University of California, San Francisco, CA, United States

## Abstract

Combination therapies targeting multiple recovery mechanisms have the potential for additive or synergistic effects, but experimental design and analyses of multimodal therapeutic trials are challenging. To address this problem, we developed a data-driven approach to integrate and analyze raw source data from separate pre-clinical studies and evaluated interactions between four treatments following traumatic brain injury. Histologic and behavioral outcomes were measured in 202 rats treated with combinations of an anti-inflammatory agent (minocycline), a neurotrophic agent (LM11A-31), and physical therapy consisting of assisted exercise with or without botulinum toxin-induced limb constraint. Data was curated and analyzed in a linked workflow involving non-linear principal component analysis followed by hypothesis testing with a linear mixed model. Results revealed significant benefits of the neurotrophic agent LM11A-31 on learning and memory outcomes after traumatic brain injury. In addition, modulations of LM11A-31 effects by co-administration of minocycline and by the type of physical therapy applied reached statistical significance. These results suggest a combinatorial effect of drug and physical therapy interventions that was not evident by univariate analysis. The study designs and analytic techniques applied here form a structured, unbiased, internally validated workflow that may be applied to other combinatorial studies, both in animals and humans.

In a typical biomedical experiment, many outcome measures are collected, but the published findings highlight only a highly-selected subgroup of these results. Univariate statistics (t-test, ANOVA, regression) are usually applied to detect significant effects, one outcome at a time^1^,^2^,^3^. However, methods for interpreting ‘significance’ across an ensemble of categorical, nominal and numeric tests are not straightforward. Meta-analyses suggest wide-spread misuse of statistical significance to construct a hypothesis and ‘story’ after the fact, leading to false inferences throughout the published literature^4^,^5^,^6^. Moreover, salient combinations of factors contributing to outcomes may be overlooked when the effects of the individual factors do not reach statistical significance. These intrinsic limitations of standard approaches substantially impact reproducibility and translational potential of findings^5^,^7^,^8^,^9^, especially with respect to single outcomes or end-points.

In the context of traumatic brain injury (TBI), no one outcome measure is likely to reflect the full complexity and diverse nature of recovery, and the true results of therapeutic interventions after TBI likely lie in the relationship between numerous outcome variables. Indeed, in recent studies of TBI and spinal cord injury, pooled multicenter and multispecies data coupled to data-driven multidimensional analysis has revealed information with significant potential for therapeutic translation, which would not have been easily identified through univariate analysis of a single end-point^10^,^11^,^12^.

Analytical problems are amplified in combinatorial studies employing multiple therapeutic approaches by the need for large sample sizes and complex statistical analysis (e.g., testing of interaction effects)^13^. The potential of combinatorial therapies to provide additive or synergistic efficacy has long been recognized; however, in the injured central nervous system the complexity of the relevant outcome measures and their analysis provide a particularly difficult challenge for developing this approach^14^.

Here we combined and analyzed data obtained from three independent studies testing combinatorial approaches for improving recovery after traumatic brain injury (TBI): an anti-inflammatory agent (minocycline), a neurotrophic agent (LM11A-31)^15^, and physical therapy. These treatment modalities were chosen because each alone has been reported to improve recovery, and because their differing modes of action provide the potential for synergistic effects. LM11A-31 directly modulates p75NTR activity and may also interfere with proNGF-p75NTR interactions^15^,^16^,^17^ resulting in decreased apoptotic and increased pro-survival signaling, and protection of neuronal processes and functions such as long-term potentiation^16^,^17^,^18^,^19^. Minocycline decreases poly(ADP-ribose) polymerase activity^20^ and caspase activation^21^, and has other effects on cell signaling that inhibit cell death and dampen the immune response, including suppression of microglial M1 (pro-inflammatory) polarization. In addition various forms of exercise, physical therapy and environmental enrichment with overlapping features (e.g., running, novel environments/activities) have been found to improve post-injury outcomes^22^,^23^,^24^,^25^ with contributing mechanisms proposed including stimulation of BDNF and other neurotrophic factor production^26^,^27^, and altered microglial CX(3)CL1 signaling^28^. Drug treatments were initiated one day following injury in order to identify effects on recovery, rather than neuroprotection, and to more closely replicate real-world treatment conditions. Similarly, physical therapy was initiated 5–7 days after injury to mimic common clinical scenarios. Data were curated into a common database aligning common data elements for use in an unbiased data-driven workflow including unsupervised non-linear principal component analysis (NL-PCA) and subsequent hypothesis testing. The results indicate synergism between the neurotrophic compound LM11A-31 and minocycline, and suggest an additional influence by the physical therapy paradigms employed. More generally, the outcomes suggest that the analytic methods used can be applied to complex data sets to reveal significant interaction patterns that are reproducible across studies and diverse outcome measures.

## Results

**Figure 1:**
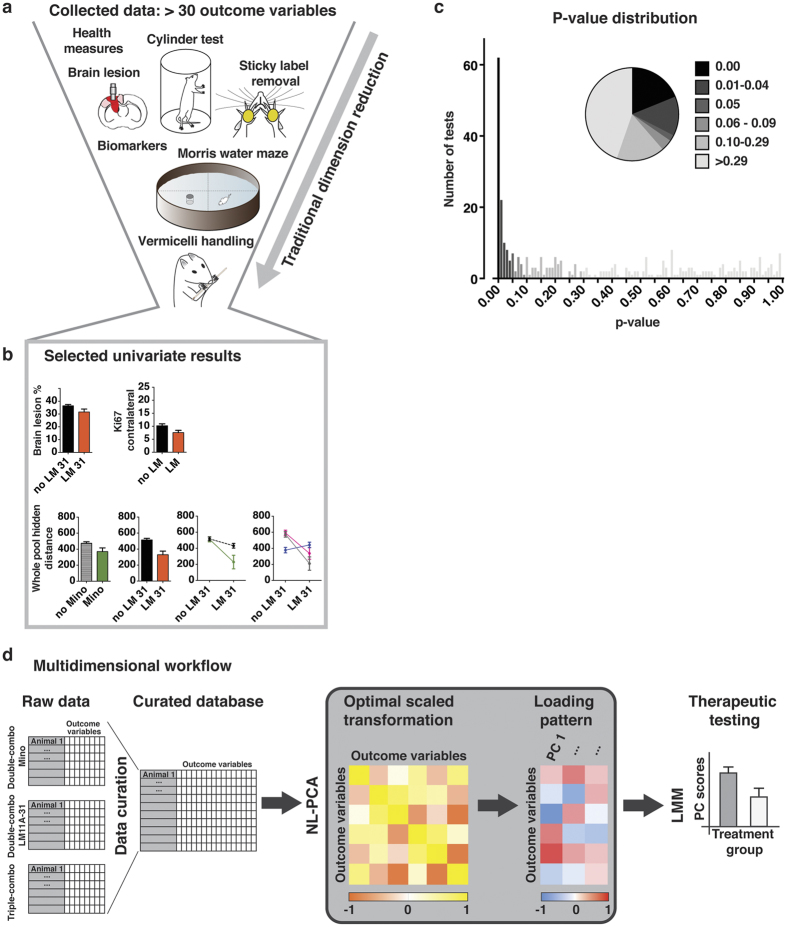
Experimental design, univariate approach and multidimensional linked analytical workflow. (**a**) Collected outcomes measures spanning information about lesion characteristics, motor, cognitive and general health domains. (**b**) Variables selected in an arbitrary fashion. Bar graphs reflect estimated marginal means of significant main effects and line graphs reflect significant interactions. (**c**) Frequency distribution and piechart of univariate p-values (**d**) Three experiments (Double-combo Mino, Double-combo LM11A-31 and Triple-combo) were cross-curated and merged into one master database. Outcome variables of all 202 rats are fed into a non-linear principal component analysis (NL-PCA). NL-PCA handles different analysis levels (e.g., ordinal and numeric) in the dataset by optimal-scaling transformations. The NL-PCA loading pattern shows the weight of every outcome variable on the obtained PCs. Individual subject-level PC scores are calculated by summing the optimally-transformed data variable values weighted by loadings. A linear mixed model (LMM) tested the effect of treatment group on the multidimensional outcome measure (i.e., PC score). Abbreviations: LM, LM11A-31; Mino, minocycline; PC, principal component.

The three studies analyzed contained an aggregate N of 202 rats, with more than 30 recorded variables per subject (Fig. 1a, Supplementary Table 1). A representative example of lesion extent and location resulting from controlled cortical impact (CCI) is shown in Supplementary Fig. 1. Standard univariate analysis of the raw source data resulted in over 6000 pair-wise differences (by t-test) and over 300 main effect and interaction tests (by ANOVA). 33% of these reached significance (Fig. 1b,c, Supplementary Table 2–3). However, selecting a set of findings to report *post hoc* exclusively from these many possible significant comparisons would lead to an interpretation biased by *a priori* expectations,. From a statistical perspective, designating any one of these findings as true runs the risk of accepting a false-positive based solely on chance (family-wise type 1 error). Applying Bonferroni’s multiple-testing correction to safeguard against spurious results, revealed that 10.3% of these tests remain significant, and the less conservative Benjamini-Hochberg method resulted in 25.4% significant test results^29^. This illustrates a problem frequently faced by practicing researchers when confronted with multiple versions of the ‘truth’: which pattern of results should be reported?

To contend with these problems we developed an unbiased, data-driven approach to capture multi-scalar outcome measures (Fig. 1d). In this method, variables across endpoints were curated and placed together into NL-PCA, in which an alternating least squares algorithm drove optimal-scaling transformations and variance-maximizing dimensionality reduction to harmonize categorical, ordinal, and numeric variables in a single non-linear framework^30^. Next, a linear mixed model (LMM) was applied to the NL-PCA outcome (PC scores) to test multidimensional therapeutic impact, free from outcome selection bias.

First, we applied this NL-PCA workflow to data combined from two studies, one involving LM11A-31 and physical therapies (double-combo LM11A-31) and the other with LM11A-31, minocycline and physical therapy treatments (triple-combo). Both of these studies included a full set of functional outcome measures, lesion severity and immunohistochemistry of cell-proliferation markers. Second, to test the reproducibility of our findings we expanded our database to include a third independent study of minocycline with rehabilitation (Double-combo Mino experiment). In comparison to the double-combo LM11A-31 and triple-combo study the double-combo mino study did not include proliferation markers and therefore the NL-PCA was solely based on functional outcome measures and brain lesion histopathology. We refer to the full multidimensional outcome space as the “syndromic space” of TBI for consistency with prior publications in neurotrauma informatics^31^,^32^.

**Figure 2:**
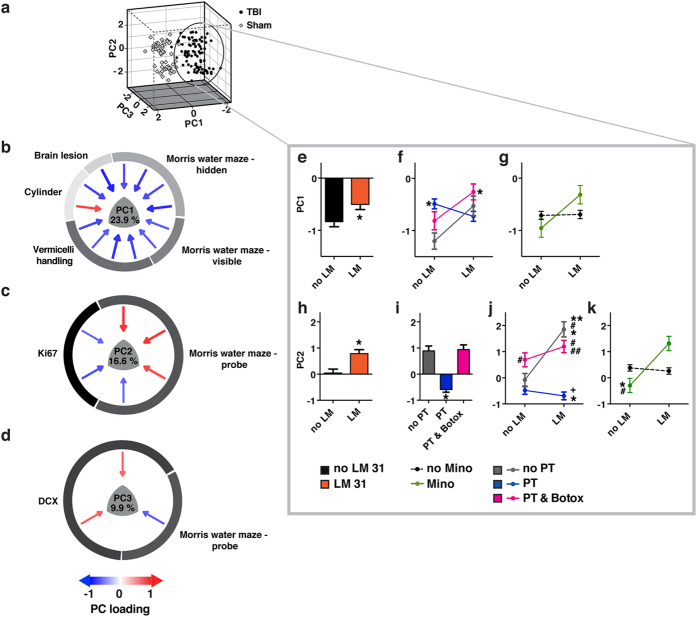
3-dimensional PCA solution: Double-combo LM11A-31 and Triple-combo experiments. (**a**) The TBI syndromic space within PC 1–3. To visualize the difference between TBI subjects and sham animals we plotted the coordinates of every animal in the newly created PC space (N = 148 animals). Two distinct subject clusters emerged, differentiating TBI and sham animals. (**b–d**) Bootstrapped, 3-dimensional NL-PCA solution loading patterns extracted across functional and brain markers. PC1 unified outcome measures that co-loaded highly with brain lesion. PC2 and PC3 captured variance in cell proliferation markers (i.e., Ki67, DCX) shared with Morris water maze probe trials. Each PC is described by variables which have loadings >|0.6| (arrows). The loading magnitude is shown by arrow width and color (blue = negative and red = positive relationship between the individual variable and the PC). The full list of loadings are listed in Supplementary Fig. 4a. (**e**–**k**) Significant main effects and interactions by linear mixed model analysis on PC scores to test the effect of drug intervention (i.e., LM and minocycline) and physical therapy on multivariate outcome (sham excluded from this analysis). Detailed statistics are listed in Supplementary Table 4. Significant pairwise posthoc Tukey’s comparisons are indicated at p < 0.05 as follows: (**f**) *Different from no LM and no physical therapy conditions. (**g**) Only significant interaction, no pairwise significance. (**i**) *PT and PT & Botox different from the PT condition. (**j**) *Different from no LM and no physical therapy conditions. ^#^Different from no LM and physical therapy conditions. ^+^Different from no LM and physical therapy & botox condition. **Different from LM treatment and no physical therapy. ^##^Different from LM treatment and physical therapy. (**k**) *Different no LM treatment and no Mino treatment. ^#^Different from LM treatment and no Mino treatment. Abbreviations: LM, LM11A-31; Mino, minocycline; PC, principal component; PT, physical therapy.

The extracted syndromic TBI space based on the double-combo LM11A-31 and triple-combo study is described and displayed in 3 dimensions to represent all measured endpoints simultaneously (Fig. 2a). Together principal components (PC) 1–3 accounted for 50.4% of the total variance. To understand how all outcomes move together as a unit and relate to markers of cellular proliferation (i.e., Ki67, doublecortin (DCX)), NL-PCA loading plots (Fig. 2b–d) as well as underlying optimal transformations and automated tests of statistical rigor were performed (Supplementary Fig. 2–3). A complete list of the loadings can be found in Supplementary Fig. 4a. Crucially, the internal (comparison of random subsamples with the same data set) and external cross-validation (between study) were performed to test the stability of the NL-PCA solution by comparing loading patterns using formal pattern matching statistics (Supplementary Fig. 4a–c). The internal cross-validation confirmed the final dimensionality (i.e., 3 PCs) that was determined by the retention rules. However, PC3 did not reliably cross-validate between experiments (external cross-validation) (Supplementary Fig. 4c). Combined, the internal and external cross-validation suggested that PC1 and PC2 represent rigorous, stable clusters of variance shared across outcomes measures and could be used to test therapeutic impact on the complete TBI syndrome. PC3 on the other hand was not carried forward in the analysis of therapeutic effect.

The effects of drug and physical therapy interventions on PC1-2 were then tested (Fig. 2e–k). Neurotrophic agent LM11A-31 increased both PC1 (p < 0.05, Fig. 2e) and PC2 (p < 0.001, Fig. 2h) (see Supplementary Table 4 for detailed statistics). For both PC1 and PC2, the effect of LM11A-31 was modulated by co-administration of minocycline (p < 0.05) and physical therapy (p < 0.05). Loading plots revealed that increased PC1 reflects therapeutic benefits on both motor and cognitive outcomes and PC2 findings suggest improved memory function specifically associated with decreased cell proliferation in the brain contralateral to the injury measured at a time point remote from the injury. Post-hoc examination of significant effects on PC1 (Fig. 2f–g) and PC2 (Fig. 2i–k) suggests that LM11A-31 may have little or no effect in animals receiving physical therapy without botulinum toxin (botox), while promoting similar improvements in the no physical therapy and physical therapy + Botox groups. The interactions between minocycline and LM11A-31 on PC1 and PC2 indicated an overall amplification by minocycline of LM11A-31 effects. It is noteworthy that in the context of factorial designs tested by LMM, main effects have higher n and therefore higher power, than interaction terms (see Supplementary Table 1). Regarding the drug effects, the results indicate a significant main effect of LM11A-31, pooled across studies (n = 46). The same cannot be said for minocycline (n = 44). Yet, the subset of subjects that received LM11A-31 and minocycline (n = 11), performed better than their specific LM11A-31-alone control group (Supplementary Tables 1–5). Further, our data suggests physical therapy in combination with Botox or no physical therapy shows more beneficial effects than physical therapy alone. These results need to be interpreted with caution, as only one of the three experiments used physical therapy without Botox and the number of animals that received the combination of Minocycline and LM11A-31 is low. Thus, even though our study shows clear drug effects pooled across experiments, the potential interaction between the pharmacotherapies alone and the combination with physical therapy is an area for future studies.

To test the reproducibility of findings we expanded our database to include an independent study of minocycline with rehabilitation (Double-combo Mino experiment). PC1 survived both the initial PC retention rules and rigorous internal and external cross-validation (Supplementary Fig. 2 and Supplementary Fig. 5a–c) Comparable to the previous analysis motor and cognitive outcomes clustered with brain lesion severity (Supplementary Fig. 5a–d). LMM results replicated the effect of LM11A-31 (p < 0.001) and its interaction with physical therapy intervention (p < 0.001) on PC1 (see Supplementary Table 5 for detailed statistics, Supplementary Fig. 5e,f). Altogether, the results suggest that the global effect of LM is substantially enhanced by combinatorial therapies.

## Discussion

Univariate testing of the 30-plus different endpoints comprising all of the measured outcomes in the studies presented here leaves uncertain which analyses are significant, and introduces a substantial risk of selection bias in choosing those which should be reported^6^,^9^. In contrast, multidimensional significance requires that robust effects endure across multiple outcome measures. Use of the data-driven approach described here revealed several robust multidimensional interactions between the treatment modalities that would not have been evident using conventional univariate analyses.

This analysis reveals several unanticipated effects and relationships between the treatment modalities. First, there was an improvement of post-TBI behaviors in response to LM11A-31 treatment delivered 24 hrs following injury when pooled across LM11A-31 combo studies, which represents a substantial extension of the treatment window into a clinically tractable range. Second, the LM11A-31 effect was modulated by minocycline, which itself had weaker effects in the global analysis. Notably, this combinatorial effect occurred across both PC1 and PC2 outcome sets, suggesting an impact on both broad lesion-correlated neurobehavioral outcomes (PC1) and more subtle cell proliferation/neurocognition outcomes (PC2). This, along with prior studies^33^, is consistent with the possibility that late-delivered LM11A-31 is weakly anti-inflammatory if at all, and requires suppression of inflammation, as provided by the more potent and directly acting minocycline to produce its protective effects. This could occur through improved neuronal survival and structural integrity, as well as enhancement of plasticity mechanisms, though may not involve significant long-term changes in neurogenesis, as univariate analysis of numbers of proliferative and neuronal precursor cells showed minimal or absent changes due to either compound. Of note, though not examined in the present experiments, prior studies showed significant long-term survival of newly generated hippocampal neurons after the injury in response to LM11A-31 treatment^19^. In addition, though minocycline may function following trauma in part by diminishing microglial production of proNGF^34^, which could potentiate LM11A-31 actions^16^,^17^, it may also decrease p75NTR expression^34^, which could have opposing effects. Thus, though the mechanisms remain unclear, the finding of a robust interaction between the two compounds with a clinical meaningful time of onset of drug administration supports further investigation of their combination. Second, there was a largely neutral to interfering interaction between the physical therapy and pharmacologic treatments. Since exercise may work at least partially through pathways involving neurotrophins and microglia, with enriched environment (EE) adding other mechanistic components, the combination of EE/Exercise and pharmacotherapy encompasses numerous potential points of interaction. Of particular note are the possibilities of aberrant/excessive and/or ill-timed promotion of increased plasticity. Several prior studies of constraint-induced movement therapy and other physical therapeutic modalities have suggested that treatment that is too early or too intense may be detrimental^35^,^36^,^37^,^38^. Further, a recent analysis of the combination of a plasticity-enhancing treatment (anti-NOGO-A antibody) with physical training following stroke in rats found that concurrent training led to overabundant and misdirected sprouting, with concomitant degradation of motor performance^39^. Though the application of physical therapeutic measures was delayed by 5–7 days in our studies, this may not have been long enough to avoid these effects, and the LM11A-31 treatment and physical therapy time frames were overlapping.

A third pattern of note is a disjunction between the effects of physical therapy with or without botox-mediated limb restraint. Whereas for several measures physical therapy + Botox treated animals tended to exhibit responses similar to those receiving no physical therapy, physical therapy -without-botox treated animals tended to diverge, beginning at or above the level of performance of physical therapy -untreated animals and declining with the addition of LM11A-31. Response differences between the PT with- and without-botox groups were further substantiated by differences observed with univariate analyses in the expression of proliferative and immature neuronal markers with physical therapy -without-botox producing a substantial bi-hemispheric increase of both markers relative to the other groups.

Together the observations from these studies, along with other published findings, suggest that future TBI studies might focus on early LM11A-31/minocycline combined pharmacotherapy followed by physical therapy (without limb restraint) initiated at a substantially delayed timepoint.

More generally, the outcomes suggest that the analytic methods used can be applied to complex data sets to reveal significant interaction patterns. This approach is most appropriate when there are multiple correlated endpoints spanning multiple domains (e.g. histology and behavior; behavior and physiology; health and behavior; or all of the above). This represents a large majority of TBI studies in the literature. Our findings demonstrate that unbiased multidimensional pattern detection can be applied to establish reproducibility across independent runs of a study^40^. The generation of these robust results in an unbiased fashion demonstrates the utility of the analytic methods applied here, and suggests the value for other complex translational studies.

The main limitation of this work is in the retrospective, ‘meta-analytic’ nature of the study. We combined and analyzed data obtained from three independent experiments, which is common in clinical research but less common in preclinical research. In this context, the results of the interaction effects need to be considered preliminary and subject to revision with additional data. For example, the drug-interacting effects of physical therapy may depend on differences between experiments, an observation that needs further prospective study for full validation. However, the LM11A-31 drug effects remained consistent across studies, despite these caveats, suggesting strong reproducibility in the context of TBI models used here.

## Materials and Methods

Experimental procedures were conducted according to the National Institute of Health Guide for the Care and Use of Laboratory Animals and were approved by the San Francisco Veterans Affairs Medical Center Animal Care and Use Committee. Male Sprague-Dawley rats, aged 8–10 weeks (250–300 g) were obtained from Simonsen Laboratories, Gilroy, CA. They were housed 2 per cage on a reverse light-dark cycle, with access to standard rodent food and water ad libitum. Surgical procedures were performed after 1–2 weeks of habituation in the animal housing facility. LM11A-31 was obtained in part from the Institute for Therapeutics Discovery & Development, University of Minnesota and the remainder was synthesized by Ricerca Biosciences, Concord, Ohio and generously provided by Dr. Frank Longo, Stanford University. All other reagents were obtained from Sigma-Aldrich, St Louis, MO, except where otherwise noted.

### Studies and data curation

Three independent experiments were performed prospectively, and the data were then retrospectively curated by independent data scientists and combined into a single database for ensemble analyses. Initial target numbers of animals for each treatment group were determined using power calculations for univariate comparisons of behavioral parameters within each of the three experiments based on estimates from prior studies in participant laboratories. Final group numbers were a function of these initial determinations, as well as logistical and practical limitations in performing the experiments. The design of the 3 independent experiments (Double-combo Mino, Double-combo LM11A-31 and Triple-combo) including the number of animals in each experimental group is presented in Supplementary Table 1. Briefly, all studies involved unilateral CCI and craniotomy-only sham controls. An anti-inflammatory agent (minocycline) and/or a neurotrophic agent (LM11A-31) were administered beginning 24 hours after the surgeries. Where used, botulinum toxin (Botox) was injected into the ipsilateral (non-paretic) forelimb 4 days after the CCI to encourage use of the paretic limb, and physical therapy was initiated 5 days after CCI. Behavioral outcome measures were assessed 10–14 weeks after CCI, and rats were euthanized for histological assessments at 14–16 weeks. As detailed in Supplementary Table 1, the treatment groups for the Double-combo Mino experiment were: (a) no treatment; (b) minocycline; (c) physical therapy plus Botox; and (d) minocycline combined with physical therapy plus Botox. The treatment groups for the Double-combo LM11A-31 experiment were: (a) no treatment; (b) LM11A-31; (c) physical therapy plus Botox; and (d) LM11A-31 combined with physical therapy plus Botox. The treatment groups for the Triple-combo experiment were: (a) physical therapy alone; (b) physical therapy plus minocycline; (c) physical therapy plus LM11A-31; and (d) physical therapy plus minocycline plus LM11A-31.

In each of these experiments, rats were arbitrarily taken for CCI or sham surgery, and animals from each group housed 2 to a cage. On post-surgical day 1, cages were serially assigned to post-operative treatment groups. Physical therapy was performed by personnel blinded to the surgical and drug-treatment conditions and the behavioral and histological assessments were likewise performed by personnel blinded to the treatment conditions. Data from the Double-combo Mino experiment was previously published^41^.

### Induction of traumatic brain injury

CCI on the left hemisphere was performed as described in d’Avila *et al*.^42^. Animals were fasted for 2 hours prior to surgery to reduce variability in blood glucose levels, and were anesthetized with ketamine/xylazine. A craniotomy was performed to expose the dural surface, and a CCI impact device (Pinpoint Precision Cortical Impactor, Hatteras Instruments, Cary, NC) was used to produce a unilateral injury using an impact tip of 5 mm in diameter with strike velocity of 1.5 m/s, strike depth of 5 mm, and dwell time of 120 ms. Sham surgery animals underwent craniotomy only. Bupivacaine analgesic was injected into the skin sutures, and the rats were placed in a warmed (35 C) recovery chamber until ambulatory. Rats which were not ambulatory within 2 hours or which developed signs of infection or distress at any point following the surgical procedures were euthanized (<5% of the subject animals).

### Drug administration

Minocycline was administered as a daily intraperitoneal injection for 14 days, 25 mg/kg in approximately 1 ml of 0.1 M phosphate buffer (pH 7.4), beginning 24 hours after CCI. LM11A-31 was administrated as a daily intraperitoneal injection for 20 days (Double-combo LM11A-31 experiment) or 21 days (Triple-combo experiment), beginning 24 hours after injury at a dose of either 50 mg/kg (Double-combo LM11A-31 experiment) or 75 mg/kg (Triple-combo experiment). Animal receiving both minocycline and LM11A-31 received a single 1 ml injection. In all cases rats not treated with drug received i.p. injections of vehicle alone.

#### Physical therapy and botox-induced limb constraint

Animals received 10 days 1-hour/day physical therapy beginning 5–7 days post-surgery over a period of 14 days. The physical therapy consisted of 10 minutes each of daily rope climbing, net traversal, and voluntary wheel running, and 30 minutes of walking in rodent balls or forced running on a rodent running wheel (Lafayette Instrument, Lafayette, IN, USA) as described previously^41^. Rats were continuously attended by research staff to ensure participation in the physical therapy regimen. Rats not given physical therapy remained in their home cages. A subgroup of the rats receiving physical therapy also had contralesional (non-paretic) forelimb restraint induced by injection of 1.25U botulinum toxin type A (Botox A) into 4 forelimb muscles^41^ on day 1–4 after surgery.

### Neurobehavioral assessment

Behavioral testing began 10–14 weeks post injury and included a battery of motor and cognitive behavioral tasks.

#### Cylinder test

Forelimb use asymmetry was measured using the cylinder test. Rats were placed individually in a Plexiglas cylinder with a height of 30 cm and a diameter of 20 cm. Uninjured rats explore the cylinder wall with equal amount of paw placements of both forelimbs whereby in unilaterally injured animal the paw placement of the affected limb is reduced. From a 10-minute videotaped session the rats paw placements on the cylinder wall during rearming were counted. Limb preference was calculated as the percent use of either forelimb during rearing^43^.

#### Sticky label removal test

During the sticky label test a round 1.25 diameter adhesive tape was placed on the plantar surface of each forepaw. The time to remove the tape from each forepaw was measured. Five trials were preformed and the median of the five trials was calculated. The maximum time allowed was 10 minutes^42^.

#### Vermicelli handling test

Skilled forepaw use was assessed with the Vermicelli handling test^44^. The rats were given 5 pieces of pasta, each 7 cm in length, in their home cage and their behavior was videotaped^41^,^42^. Typical handling of the vermicelli involves holding the pasta asymmetrical with paws, one paw guiding and one paw grasping. The paws typically remain apart until the pasta reaches half in length. Atypical handling behaviors were classified into two categories (i.e., Type I and II). Type I atypical handling behavior included (i) symmetrical paw placement, (ii) hunched body position, and (iii) head tilted or face lowered while eating. The occurrence of Type I atypical behaviors were scored as 1 if the behavior occurred and as 0 if not. The Type II atypical behaviors included (i) mouth pulling, (ii) single paw use, (iii) switching the guide and the grasp paw, and (iiii) flipping or dropping the pasta during eating. The frequencies of occurrence of the Type II atypical behaviors were summed.

#### Morris water maze test

Spatial learning and memory was assessed with the Morris water maze test^45^. The rats’ ability to locate a submerged platform (10 cm diameter, 2.5 cm below the water surface) was measured in a circular pool (180 cm diameter, 60 cm depth). Rats were trained to locate a visible platform during the first day (indicated by a flag hanging above the platform), followed by the hidden-platform training for additional 4–7 days. During both the visible and hidden platform training, 6 daily trials, counter-balanced for distance and drop locations, were performed. Latency and the distance to locate the platform and swim velocity were measured via a video tracking system (Ethovision, Noldus Information Technology, Sterling, VA). Data from 4 days of hidden platform training were included for PCA analysis. One day after the completion of the hidden platform training, rats were subjected to the last trial in which the hidden platform was removed to assess retention memory (i.e., the probe trial). An annulus with a diameter of 20 cm was defined concentric with the previous location of the hidden platform. In the probe trial, the time spent, the number of crossings and the latency of the first crossing were measured for the annulus, the target quadrant and the platform area. In all sessions (i.e., visible platform, hidden platform and probe trial) thigmotaxic behavior, swimming within 20 cm of the edge of the pool, was determined^41^.

### Immunohistochemistry

Rats were anesthetized and transcardially perfused with 4% formaldehyde. Brains were removed and post-fixed in 4% formaldehyde overnight, cyroprotected in sucrose, and cryostat sectioned into 40 μm coronal sections. Immunostaining was performed on 40 μm thick free-floating sections using the following antibodies: anti-NeuN, Chemicon mouse NB110-40766SS; anti-Ki67, Abcam rabbit ab16667 lot#GR129115-1; anti-doublecortin, goat sc-8066 lot#A3007. NeuN labeling was visualized with fluorescently labeled anti-mouse IgG. Immunostaining for Ki67 and doublecortin were done simultaneously and visualized using fluorescently tagged anti-rabbit IgG and anti-goat IgG, respectively. Cell nuclei were labeled with DAPI. Controls prepared in the absence of primary or secondary antibodies showed no signal.

### Assessment of lesion volume

The TBI lesion cavity was determined as previously described by quantifying the volume devoid of neurons^46^. Volume analysis for each brain used seven evenly spaced Neu-N stained sections that included the anterior and posterior margins of the lesions.

### Assessment of hippocampal cell proliferation

Ki67 was used as a marker for proliferating cells, and doublecortin (DCX) as a marker for dividing neuronal precursor cells and newly formed neurons. Each group of sections to be compared was stained at the same time, and all sections were and photographed under identical conditions. Immunostaining, photography, and cell counts were performed by individuals blinded to the treatment conditions. Two sections were analyzed from each brain; one from the posterior hippocampus and one from the anterior hippocampus. On each section, the total number of cells positive for each marker (Ki67 and DCX) was counted in the hippocampal dentate gyrus ipsilateral to the CCI, and separately counted in the dentate gyrus contralateral to the CCI. The values obtained in the two sections were summed for each brain, and the summed values were averaged among the brains in each treatment group.

### Statistical analysis

Statistical analyses were performed using SPSS Statistics 23 (IBM). We performed three distinct analyses. First, we performed traditional univariate analyses of all outcome measures of interest. We tested the main effect and interactions of drug (i.e., minocycline and LM11A-31) and physical therapy intervention (i.e., no physical therapy, physical therapy alone and physical therapy in combination with botox). For linear variables a LMM was applied^47^. Count data were assessed by a generalized linear model (Poisson probability distribution, log link function). To test how many of the univariate effects would survive multiple-testing correction we used Bonferroni correction and the Benjamini-Hochberg method^29^. Second, to remain sensitive to multimodal changes induced by the combinatorial interventions and to use the full set of outcome measures, an unbiased data-driven multivariate approach was applied. The data-driven analysis was performed without prior assumptions; therefore a primary outcome measure was not defined. An independent analyst blinded to the experimental condition performed all data-driven analyses. Third, after multidimensional cross-validation of outcome patterns experimental conditions were decoded for explicit hypothesis testing using a LMM on PCA derived scores (PCA-LMM) (Fig. 1d). The interaction effects (i.e., synergistic and counteracting) of different drug interventions and physical therapies were all assessed using this linked analytical workflow.

To integrate data across outcome measures with diverse scales we used a non-linear PCA (NL-PCA)^30^,^48^. A tutorial on how to apply NL-PCA has been provided by Linting *et al*.^48^ and the SPSS syntax for the current NL-PCA is available as Supplementary Note 1. NL-PCA is suitable for variables of mixed measurement levels (nominal, ordinal and numeric). In NL-PCA variables are assigned numerical values through a process called optimal scaling transformation. Optimal scaling assigns quantitative values to categorical variables in an optimal way, meaning maximizing the variance of the predefined number of PC (i.e., dimensions). The NL-PCA dimension reduction and stability testing workflow is shown in Supplementary Fig. 2. NL-PCA was initially applied using a 6-dimensional solution. The final dimensionality of the PCA (i.e., number of principal components) was defined based on the following criteria: (1) Kaiser rule, retaining principal components with eigenvalues >1; (2) Cattell rule, retaining principal components above the elbow in the scree plot; (3) PC over-determination, retaining components with at least four loadings above 0.6^49^,^50^,^51^. Based on these criteria, the final number of PC were defined to maximize the variance accounted for and at the same time reducing the number of components (Supplementary Fig. 2a). In NL-PCA the selected dimensionality affects analysis results. Therefore, after determining the number of PC to retain the PCA was re-run with the final dimensionality.

Stability of PCA solutions was further assessed by internal and external cross-validation (Supplementary Fig. 2b). To determine the internal stability of the NL-PCA solution, we performed nonparametric balanced bootstrapping procedure using 2000 iterations and Procrustes rotation^52^. The non-bootstrapped PCA solutions assessed for cross-validation against the bootstrapped PCA solution by using pattern matching statistics: root mean square difference in PC loading patterns, the coefficient of congruence, the Pearson product moment correlation coefficient, and the Cattell salient variable similarity index^53^,^54^. Convergence of these metrics indicates consensus of highly-reproducible PC patterns^53^. Though there exist no clear threshold values for the root mean square and the coefficient of congruence, it has been suggested that root mean square values approaching 0.0 and coefficient of congruence approaching 1.0 indicate an appropriate fit of both the magnitude and the sign of the loading patterns. In the current study, PC loading pattern matching was set at p < 0.05 for both Pearson r and the salient variable similarity index (s). For the salient variable similarity index, we used the conservative cutoff of |.4| for assessing salient loading^51^,^54^. For the external cross-validation the dataset was split up into its separate experiments (i.e., Double-combo Mino, Double-combo LM11A-31 and Triple-combo). PCA was then run on each experiment separately and the resulting PCA solutions were compared using the same pattern matching statistics as for the internal cross-validation.

All NL-PCA were performed in and unsupervised manner by an analyst who was blind to experimental condition. Only after cross-validation were experimental conditions decoded for explicit hypothesis testing. Hypothesis testing LMM was used on the stable PC (based on internal and external cross-validation) to test the effect of drug intervention and physical therapy intervention (i.e., no physical therapy, physical therapy alone and physical therapy in combination with botox) on principal component scores, thereby assessing the full set of functional outcome measures and brain markers simultaneously (Supplementary Fig. 2c). Significant effects were followed by Tukey’s *post hoc* test of group means. Statistical significance for all analysis was set at α = 0.05.

## Additional Information

**How to cite this article:** Haefeli, J. *et al*. A data-driven approach for evaluating multi-modal therapy in traumatic brain injury. *Sci. Rep.* **7**, 42474; doi: 10.1038/srep42474 (2017).

**Publisher's note:** Springer Nature remains neutral with regard to jurisdictional claims in published maps and institutional affiliations.

## Supplementary Material

Supplementary Information
