# Supplementary material for: A data-driven approach for evaluating multi-modal therapy in traumatic brain injury
Source: Sci Rep. 2017 Feb 16;7:42474. doi: 10.1038/srep42474 (PMC5311970; doi:10.1038/srep42474)
Supplement: Supplementary Information [file srep42474-s1.pdf]

A data-driven approach for evaluating multi-modal therapy in traumatic brain injury

Jenny Haefeli[1*], Adam R. Ferguson[1,2*], Deborah Bingham[2], Adrienne Orr[2], Seok Joon Won[2,3], Tina I. Lam[2,3], Jian Shi[2,3], Sarah Hawley[2], Jialing Liu[1,2], Raymond A. Swanson[2,3], Stephen M. Massa[2,3]

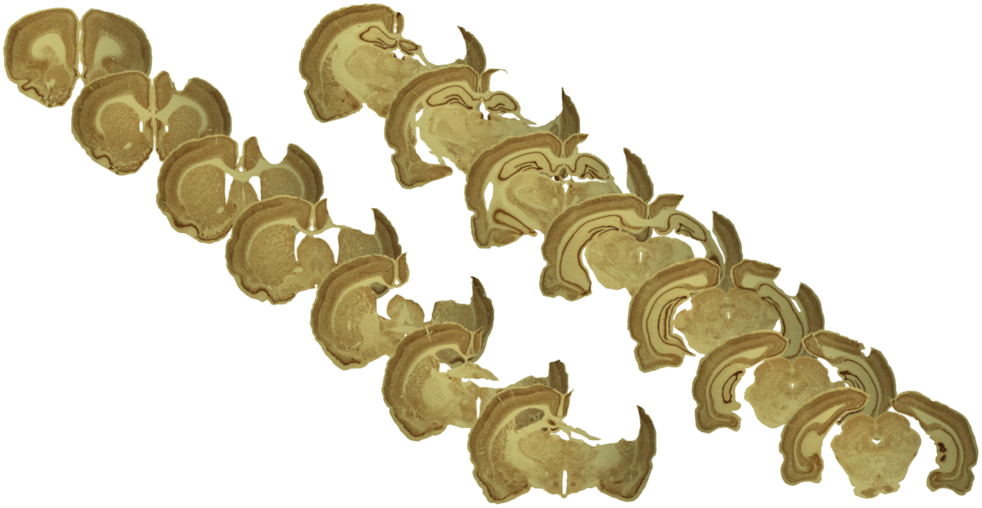

Supplementary Fig. 1

Supplementary Fig. 1 Coronal sections from a rat brain harvested four months after injury show the location and size of the injury resulting from the controlled cortical impact.

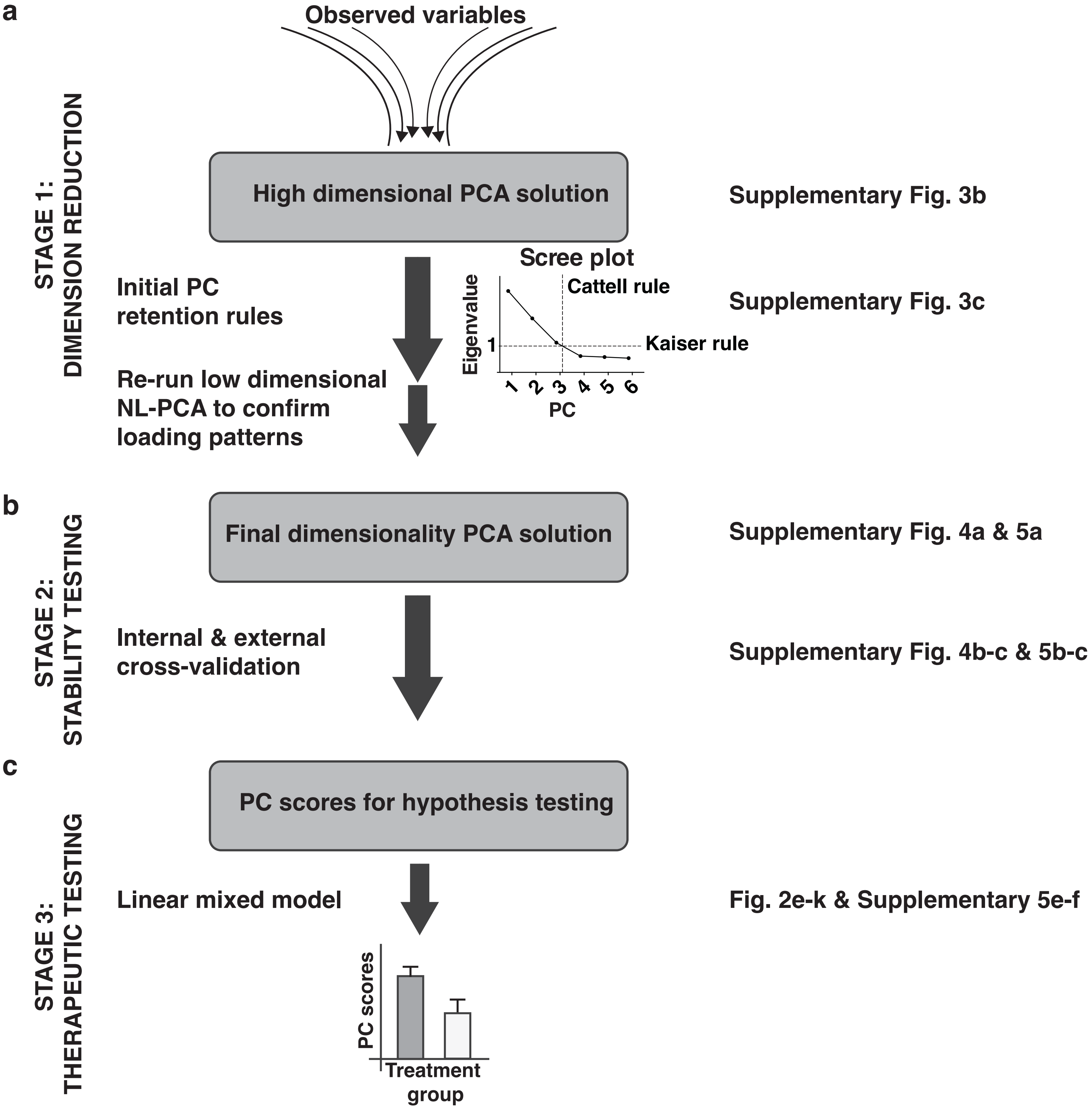

Supplementary Fig. 2

Supplementary Fig. 2 Unbiased analytical workflow. **a)** Stage 1: Dimension reduction. The observed variables were initially fed into a NL-PCA using a pre-specified 6-dimensional solution (i.e., 6 principal components). Based on initial retention rules (Scree plot and Kaiser rule) we determined the cut-offs for interpreting PCs further (e.g., PC1-3). In accordance with the SPSS manufacturers recommendation, after determining the number of PC to retain, we re-ran the NL-PCA with specifying final dimensionality a prior to confirm the PC loading structure. This work-flow, is conceptually analogous to confirmatory factor analysis, where the number of factors is predefined. **b)** Stage 2: Stability Testing. Stability of the final PCA solution was assessed by using internal and external cross-validation prior to defining PC scores as the multidimensional outcome for hypothesis testing. For the internal cross-validation we used bootstrapping (2000 iterations of random subsampling with replacement). The non-bootstrapped PCA solution was assessed for cross-validation against the bootstrapped PCA solution by using pattern-matching statistics to compare the extracted PC loading patterns. For the external cross-validation the dataset was split up into its separate experiments. PCA was then run on each experiment separately and the resulting PCA solutions were compared using the same pattern matching statistics as for the internal cross-validation. **c)** Stage 3: Hypothesis testing. PC scores that showed stable loading patterns in both the internal and external cross-validation were used as syndromic outcome measures in therapeutic

hypothesis testing. Abbreviations: PC, principal component; NL-PCA, non-linear principal component analysis.

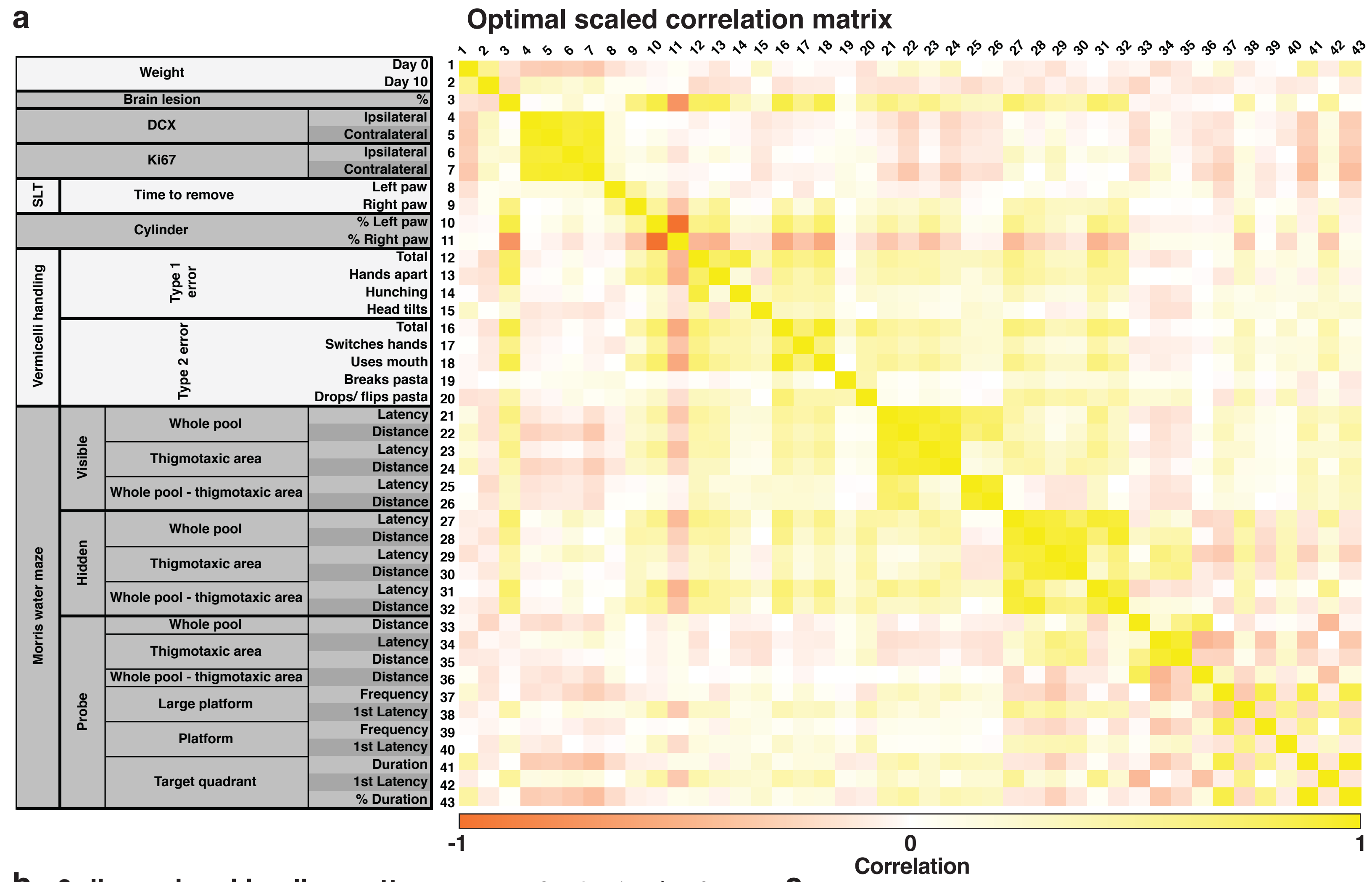

b 6-dimensional loading pattern

Outcome variables

#	Variable	PC 1	PC 2	PC 3	PC 4	PC 5	PC 6
1	Weight – Day 0	0.08	0.45	-0.13	-0.24	-0.10	-0.22
2	Weight – Day 10	0.24	-0.17	0.28	-0.22	-0.05	-0.17
3	Brain lesion – %	-0.86	0.00	0.17	0.16	0.01	0.10
4	DCX – Ipsilateral	0.11	-0.57	0.62	-0.01	0.30	-0.09
5	DCX – Contralateral	0.04	-0.55	0.64	-0.02	0.32	-0.10
6	Ki67 – Ipsilateral	-0.12	-0.61	0.53	0.05	0.28	-0.09
7	Ki67 – Contralateral	0.04	-0.69	0.55	0.00	0.23	-0.08
8	SLT – Time to remove – Left paw	-0.05	-0.28	0.07	0.26	0.05	0.38
9	SLT – Time to remove – Right paw	-0.47	-0.13	0.07	0.20	0.10	0.18
10	Cylinder – % Left paw	-0.73	-0.06	0.25	0.13	-0.10	0.10
11	Cylinder – % Right paw	0.73	0.07	-0.25	-0.13	0.10	-0.10
12	Vermicelli handling – Type 1 error – Total	-0.72	0.15	0.17	0.20	-0.15	-0.04
13	Vermicelli handling – Type 1 error – Hands apart	-0.64	-0.08	0.05	0.14	0.09	0.23
14	Vermicelli handling – Type 1 error – Hunching	-0.39	0.13	0.15	0.20	-0.20	-0.23
15	Vermicelli handling – Type 1 error – Head tilts	-0.20	0.30	0.12	0.03	-0.34	-0.15
16	Vermicelli handling – Type 2 error – Total	-0.79	0.08	0.03	0.24	-0.16	-0.07
17	Vermicelli handling – Type 2 error – Switches hands	-0.52	0.17	0.13	0.07	-0.20	-0.13
18	Vermicelli handling – Type 2 error – Uses mouth	-0.75	0.12	0.14	0.20	-0.13	-0.06
19	Vermicelli handling – Type 2 error – Breaks pasta	-0.16	-0.14	-0.05	0.07	0.02	-0.03
20	Vermicelli handling – Type 2 error – Drops/ flips pasta	-0.43	-0.21	-0.22	0.25	-0.13	-0.04
21	Morris water maze – Visible – Whole pool – Latency	-0.64	0.48	0.09	-0.33	0.28	0.17
22	Morris water maze – Visible – Whole pool – Distance	-0.57	0.56	-0.10	-0.32	0.30	0.16
23	Morris water maze – Visible – Thigmotaxic area – Latency	-0.64	0.33	0.03	-0.25	0.37	-0.02
24	Morris water maze – Visible – Thigmotaxic area – Distance	-0.54	0.43	-0.22	-0.23	0.39	-0.08
25	Morris water maze – Visible – Whole pool - thigmotaxic area – Latency	-0.26	0.45	0.14	-0.26	-0.06	0.39
26	Morris water maze – Visible – Whole pool - thigmotaxic area – Distance	-0.29	0.44	0.15	-0.27	-0.01	0.44
27	Morris water maze – Hidden – Whole pool – Latency	-0.82	-0.31	-0.10	-0.14	0.05	-0.19
28	Morris water maze – Hidden – Whole pool – Distance	-0.70	-0.34	-0.44	0.06	0.15	-0.15
29	Morris water maze – Hidden – Thigmotaxic area – Latency	-0.54	-0.55	-0.36	-0.16	0.12	-0.22
30	Morris water maze – Hidden – Thigmotaxic area – Distance	-0.51	-0.35	-0.56	-0.07	0.17	-0.24
31	Morris water maze – Hidden – Whole pool - thigmotaxic area – Latency	-0.77	0.01	0.17	-0.07	-0.04	-0.10
32	Morris water maze – Hidden – Whole pool - thigmotaxic area – Distance	-0.70	-0.24	-0.24	0.15	0.08	-0.03
33	Morris water maze – Probe – Whole pool – Distance	0.09	-0.02	-0.60	0.50	0.24	0.19
34	Morris water maze – Probe – Thigmotaxic area – Latency	0.00	-0.60	-0.47	-0.27	-0.01	0.00
35	Morris water maze – Probe – Thigmotaxic area – Distance	-0.01	-0.43	-0.65	-0.09	0.02	0.02
36	Morris water maze – Probe – Whole pool - thigmotaxic area – Distance	0.11	0.34	-0.13	0.63	0.25	0.19
37	Morris water maze – Probe – Large platform – Frequency	0.07	0.72	0.13	0.17	0.18	-0.28
38	Morris water maze – Probe – Large platform – 1st Latency	-0.50	-0.30	0.00	-0.23	-0.27	0.09
39	Morris water maze – Probe – Platform – Frequency	0.06	0.47	0.24	0.28	0.13	-0.26
40	Morris water maze – Probe – Platform – 1st Latency	-0.32	-0.18	-0.24	-0.09	-0.30	-0.01
41	Morris water maze – Probe – Target quadrant – Duration	0.00	0.80	0.00	0.01	0.07	-0.26
42	Morris water maze – Probe – Target quadrant – 1st Latency	-0.48	-0.14	0.34	-0.29	-0.31	0.00
43	Morris water maze – Probe – Target quadrant – % Duration	-0.01	0.80	0.00	0.01	0.08	-0.25

-1 0 1
PC loading

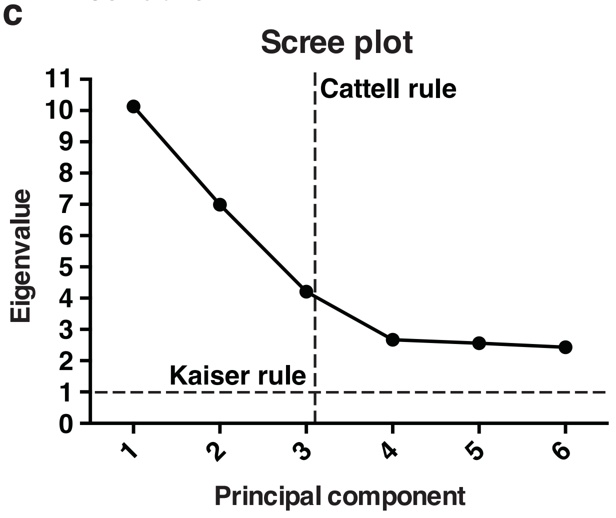

Supplementary Fig. 3

Supplementary Fig. 3 6-dimensional PCA solution: Double-combo LM11A-31 and Triple-combo experiments**. a)** Optimally-scaled correlation matrix of the 6-dimensional PCA solutions shows the bivariate association between all the outcome variables. **b)** Bootstrapped 6-dimensional NL-PCA solution loading patterns. PC1-3 had high loadings of outcome variables. **c)** Shows the scree plot for the 6-dimensional PCA solution. The Cattell and the Kaiser rules were used to define the final dimensionality of the PCA solution. The PC retention rules converged on a 3-dimensional solution; the results of the 3-dimensional solution are shown in **Fig. 2**. Abbreviations: PC, principal component; SLT, sticky label removal test.

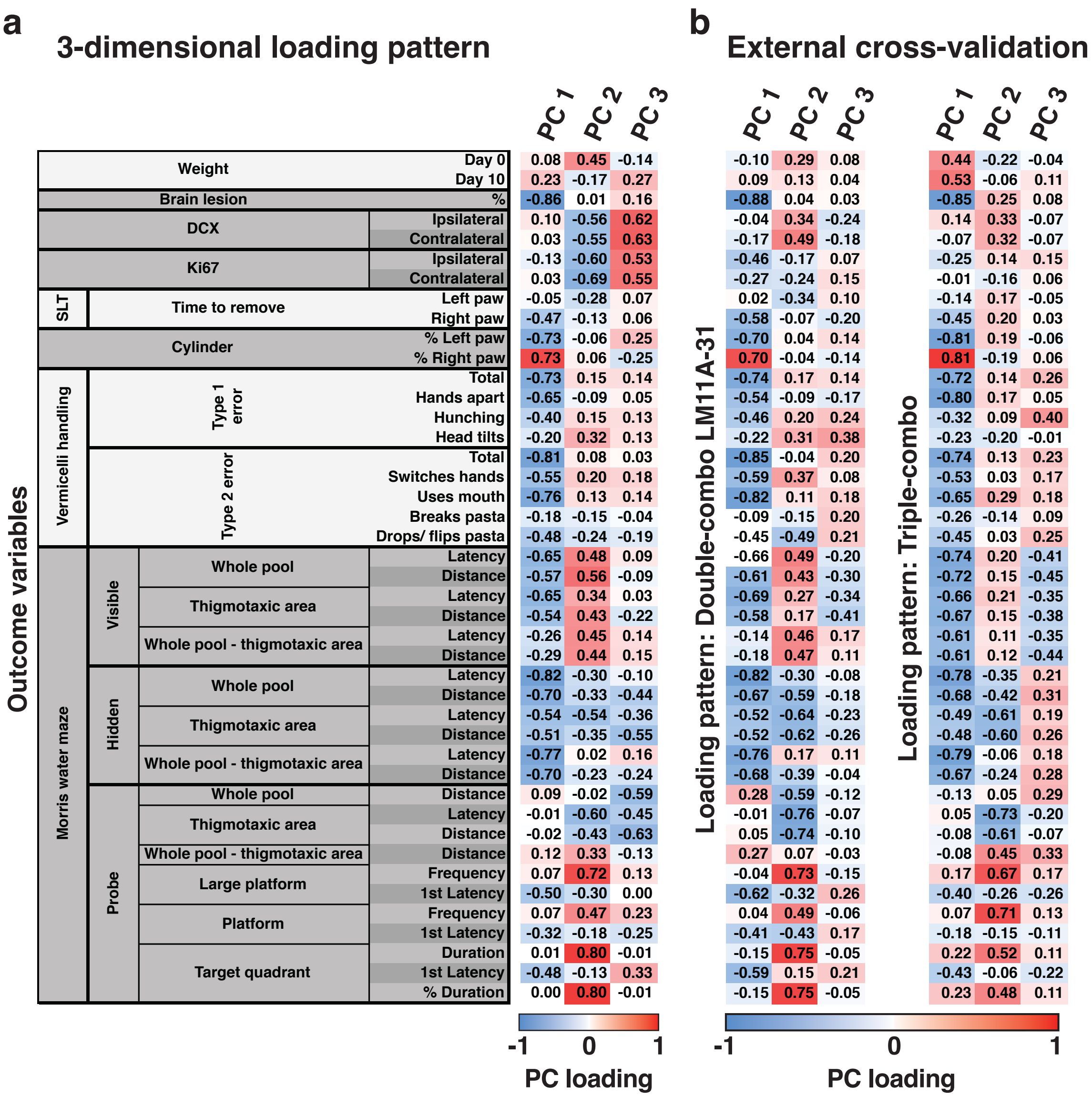

c Statistical comparison

Double-combo LM11A-31 vs. Triple-combo

	PC1	PC2	PC3
r	0.85 **	0.77 **	0.19
RMS difference	0.04	0.06	0.06
CC	0.91	0.77	0.18
s	0.80 **	0.70 **	0.00

Supplementary Fig. 4

Supplementary Fig. 4 3-dimensional PCA solution and external cross-validation: Double-combo LM11A-31 and Triple-combo experiments**. a)** Bootstrapped 3-dimensional NL-PCA solution loading patterns extracted from all outcome measures and brain markers. The color-coded representation highlights correlation between each outcome variable and the corresponding PC (blue indicating a negative and red a positive relationship between the individual variable and the PC). Internal cross-validation by pattern comparison between the bootstrapped and non-bootstrapped PCA analysis, indicated that our loading patterns were very stable (PC1, r=1.00, salient similarity index=1.00, p<0.001; PC2, r=1.00, salient similarity index=0.96, p<0.001; PC3, r=1.00, salient similarity index=0.94, p<0.001) **b)** For the external cross-validation the PCA were done for both experiments separately (Double-combo LM11A-31 and Triple-combo). The first loading pattern is based on the Double-combo LM11A-31 experiment and the second loading pattern is based on the Triple-combo experiment. **c)** Shows the statistical pattern matching comparison of the two separate PCA solutions (Double-combo LM11A-31 and Triple-combo experiments). Both PC1 and 2 show strong replication whereby PC3 did not cross-validate. This indicates that PC1 and 2 represent robust clusters of variance shared across outcomes measures. ** Indicates statistical significant (p<0.5) cross-validation. Abbreviations: CC, coefficient of congruence, PC, principal component; r, Pearson's r, RMS, root mean square, s, Cattel's s; SLT, sticky label removal test.

a 2-dimensional loading pattern

b External crossvalidation

Outcome variables; columns: PC loading (colour scale -1, 0, 1)

Outcome variables				a: PC 1	a: PC 2	b: Loading pattern: Double-combo Mino PC 1	PC 2	b: Loading pattern: Double-combo LM11A-31 PC 1	PC 2	b: Loading pattern: Triple-combo PC 1	PC 2
Weight			Day 0	0.08	0.25	-0.53	-0.04	-0.12	0.33	0.45	-0.26
			Day 10	0.21	0.04	0.01	0.07	0.07	0.16	0.54	-0.11
Brain lesion			%	-0.87	0.08	-0.87	0.00	-0.87	0.04	-0.85	0.24
SLT	Time to remove		Left paw	0.11	-0.13	0.29	0.13	0.03	-0.32	-0.15	0.17
			Right paw	-0.39	-0.03	-0.38	0.25	-0.59	-0.08	-0.45	0.21
Cylinder			% Left paw	-0.71	0.06	-0.72	0.10	-0.69	0.05	-0.81	0.19
			% Right paw	0.71	-0.06	0.72	-0.10	0.69	-0.05	0.81	-0.19
Vermicelli handling	Type 1 error		Total	-0.76	0.18	-0.85	0.15	-0.75	0.15	-0.74	0.11
			Hands apart	-0.64	-0.01	-0.72	-0.09	-0.54	-0.12	-0.80	0.15
			Hunching	-0.41	0.20	-0.49	0.50	-0.49	0.21	-0.35	0.06
			Head tilts	-0.46	0.18	-0.69	0.04	-0.23	0.35	-0.24	-0.16
	Type 2 error		Total	-0.64	0.13	-0.72	0.05	-0.86	-0.04	-0.74	0.11
			Switches hands	-0.46	0.20	-0.52	0.24	-0.58	0.37	-0.53	0.05
			Uses mouth	-0.61	0.24	-0.61	-0.28	-0.82	0.11	-0.65	0.28
			Breaks pasta	-0.11	-0.14	-0.10	0.17	-0.11	-0.16	-0.26	-0.13
			Drops/ flips pasta	-0.33	-0.21	-0.28	0.36	-0.50	-0.47	-0.48	0.08
Morris water maze	Visible	Whole pool	Latency	-0.75	0.34	-0.85	-0.10	-0.67	0.47	-0.75	0.22
			Distance	-0.71	0.34	-0.85	-0.07	-0.62	0.40	-0.74	0.16
		Thigmotaxic area	Latency	-0.68	0.25	-0.72	0.47	-0.71	0.24	-0.67	0.22
			Distance	-0.62	0.26	-0.73	0.48	-0.60	0.14	-0.68	0.15
		Whole pool - thigmotaxic area	Latency	-0.33	0.24	-0.28	-0.69	-0.13	0.46	-0.62	0.14
			Distance	-0.40	0.22	-0.41	-0.56	-0.18	0.46	-0.63	0.14
	Hidden	Whole pool	Latency	-0.85	-0.23	-0.89	-0.19	-0.83	-0.32	-0.77	-0.37
			Distance	-0.80	-0.28	-0.91	-0.18	-0.67	-0.61	-0.67	-0.45
		Thigmotaxic area	Latency	-0.55	-0.41	-0.58	0.58	-0.53	-0.67	-0.48	-0.62
			Distance	-0.55	-0.32	-0.61	0.58	-0.52	-0.65	-0.46	-0.62
		Whole pool - thigmotaxic area	Latency	-0.71	0.00	-0.65	-0.59	-0.77	0.18	-0.78	-0.08
			Distance	-0.70	-0.18	-0.76	-0.49	-0.68	-0.39	-0.66	-0.27
	Probe	Whole pool	Distance	-0.05	-0.18	-0.28	0.08	0.30	-0.59	-0.13	0.03
		Thigmotaxic area	Latency	-0.06	-0.61	-0.30	-0.50	0.00	-0.78	0.04	-0.68
			Distance	-0.06	-0.51	-0.27	-0.51	0.06	-0.76	-0.09	-0.57
		Whole pool - thigmotaxic area	Distance	-0.01	0.21	-0.11	0.40	0.28	0.08	-0.07	0.39
		Large platform	Frequency	0.21	0.73	0.51	0.10	-0.05	0.71	0.18	0.64
			1st Latency	-0.51	-0.33	-0.50	-0.09	-0.61	-0.30	-0.41	-0.24
		Platform	Frequency	0.19	0.56	0.45	0.08	0.03	0.48	0.08	0.69
			1st Latency	-0.23	-0.24	-0.06	0.01	-0.39	-0.42	-0.19	-0.12
		Target quadrant	Duration	0.11	0.70	0.26	-0.06	-0.17	0.76	0.22	0.49
			1st Latency	-0.43	-0.04	-0.36	0.13	-0.60	0.14	-0.43	-0.06
			% Duration	0.11	0.72	0.30	0.04	-0.17	0.76	0.23	0.44

PC loading: -1 0 1

c Statistical comparison

Double-combo MIno vs. Double-combo LM11A-31

	PC1	PC2
r	0.79 **	-0.02
RMS difference	0.05	0.22
CC	0.89	-0.01
s	0.87 **	-0.16

Double-combo Mino vs. Triple-combo

	PC1	PC2
r	0.82 **	0.14
RMS difference	0.05	0.14
CC	0.90	0.14
s	0.78 **	0.00

Double-combo LM11A-31 vs. Triple-combo

	PC1	PC2
r	0.84 **	0.76 **
RMS difference	0.04	0.06
CC	0.91	0.76
s	0.87 **	0.67 **

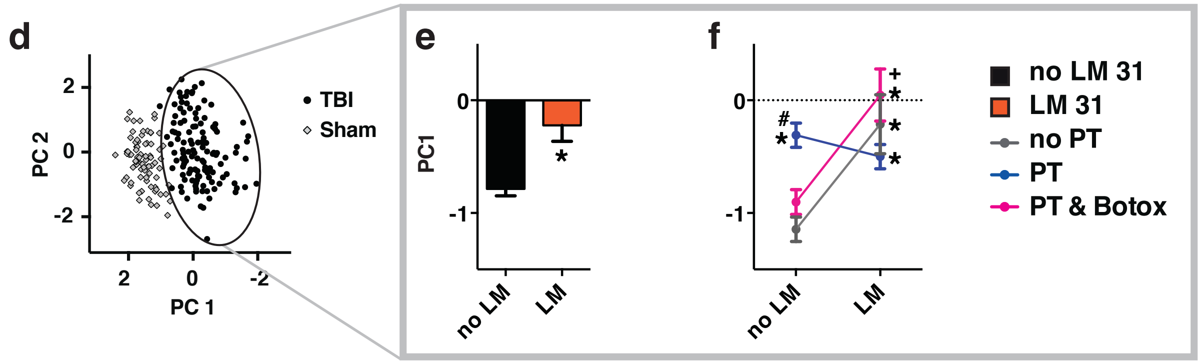

Supplementary Fig. 5

Supplementary Fig. 5 2-dimensional PCA solution and external cross-validation: all experiments combined (Double-combo Mino, Double-combo LM11A-31 and Triple-combo experiments**.). a)** Bootstrapped 2-dimensional NL-PCA solution loading patterns extracted from all outcome measures. The color-coded representation highlights correlation between each individual outcome variable and the corresponding PC. Internal cross-validation by pattern comparison between the bootstrapped and non-bootstrapped PCA analysis, indicated that our loading patterns were very stable (PC1, r=1.00, salient similarity index=1.00, p<0.001; PC2, r=1.00, salient similarity index=1.00, p<0.001) **b)** For the external cross-validation the PCA were done for all experiments separately (Double-combo Mino, Double-combo LM11A-31 and Triple-combo experiments). **c)** Shows the pair-wise statistical pattern matching comparison of all separate PCA solutions. PC1 shows strong replication in the entire pattern matching comparison. PC 2 did only replicate if experiment Double-combo LM11A-31 and Triple-combo were compared to each other. ** Indicates statistical significant (p<0.5) cross-validation. **d)** Bi-plot of individual subject's PC scores in the PC1 and PC2 space (N=202 animals) of the combined PCA (all experiments combined). The PC1 and PC2 space separates the cluster of the sham animals from the cluster of the TBI animals. **e) and f)** Results of the LMM analysis of the PC 1 score to test the effect of drug intervention (i.e., LM and minocycline) and physical therapy. Detailed statistics are listed in **Supplementary Table 5.** The effects of drug and PT intervention were only tested for PC1 as based on the pattern matching statistics PC 2 was not stable throughout all 3 experiments. The sham animals were excluded for this analysis step for focused

hypothesis testing of therapeutic effects. Significant pairwise comparisons are indicated with symbols. **f)** *Different from no LM treatment and no physical therapy. #Different from the condition with no LM treatment and physical therapy. +Different from the condition with no LM treatment and physical therapy & botox. Abbreviations: CC, coefficient of congruence, LM, LM11A-31; Mino, minocycline; PC, principal component; PT, physical therapy; r, Pearson's r, RMS, root mean square, s, Cattell's s; SLT, sticky label removal test.

Supplementary Table 1: Experimental design of the three independent experiments

**Double-combo Mino**					
Treatment Group	n	Botox	Physical therapy	Minocycline	LM11A-31
CCI	6				
CCI/mino	8			**X**	
CCI/Botox/PT	8	**X**	**X**		
CCI/mino/Botox/PT	8	**X**	**X**	**X**	
Sham	6				
Sham/mino	6			**X**	
Sham/Botox/PT	6	**X**	**X**		
Sham/mino/Botox/PT	6	**X**	**X**	**X**	
**Double-combo LM11A-31**					
Treatment Group	n	Botox	Physical therapy	Minocycline	LM11A-31
CCI	14				
CCI/LM11A-31	8				**X**
CCI/Botox/PT	10	**X**	**X**		
CCI/LM11A-31/ Botox/PT	15	**X**	**X**		**X**
Sham	8				
Sham/ LM11A-31	6				**X**
Sham/Botox/PT	9	**X**	**X**		
Sham/ LM11A-31/Botox/PT	9	**X**	**X**		**X**
**Triple-combo**					
Treatment Group	n	Botox	Physical therapy	Minocycline	LM11A-31
CCI/ PT	12		**X**		
CCI/Mino/PT	11		**X**	**X**	
CCI/LM11A-31/PT	12		**X**		**X**
CCI/Mino/LM11A-31/PT	11		**X**	**X**	**X**
Sham/PT	6		**X**		
Sham/Mino/PT	6		**X**	**X**	
Sham/LM11A-31/PT	5		**X**		**X**
Sham/Mino/LM11A-31/PT	6		**X**	**X**	**X**

CCI – controlled cortical impact; Sham - craniotomy only; PT - Physical therapy; Botox – injected into ipsilateral (nonparetic) forelimb; PT - Physical therapy;

Double-combo Mino: Mino - 25 mg/kg minocycline i.p. x 14 days, first dose at 24 hours after surgery.

Double-combo LM11A-31: LM11A-31 - 50 mg/kg i.p. x 20 days, first dose at 24 hours after surgery.

Triple-combo: Mino - 25 mg/kg minocycline i.p. x 14 days, first dose at 24 hours after surgery; LM11A-31 - 75 mg/kg i.p. x 21 days, first dose at 24 hours after surgery.

Supplementary Table 2: Univariate effects – all experiments combined

		Effect	F or Wald Chi-Square	Sig.
	**Brain lesion %**	**Mino**	5.08	0.03
		**LM11A-31**	3.97	0.05
		**PT intervention**	0.95	0.39
		**Mino * LM11A-31**	0.01	0.94
		**Mino * PT intervention**	0.77	0.47
		**LM11A-31 * PT intervention**	0.80	0.45
	**Cylinder % right paw**	**Mino**	0.01	0.93
		**LM11A-31**	0.01	0.91
		**PT intervention**	9.38	0.00
		**Mino * LM11A-31**	0.04	0.83
		**Mino * PT intervention**	0.80	0.45
		**LM11A-31 * PT intervention**	0.41	0.66
**Vermicelli handling test**	**Type 1 error total**	**Mino**	0.02	0.90
		**LM11A-31**	0.00	0.96
		**PT intervention**	11.49	0.00
		**Mino * LM11A-31**	0.05	0.83
		**Mino * PT intervention**	12.87	0.00
		**LM11A-31 * PT intervention**	3.23	0.20
	**Type 1 error hands apart**	**Mino**	0.25	0.61
		**LM11A-31**	0.04	0.85
		**PT intervention**	0.17	0.92
		**Mino * LM11A-31**	0.00	0.95
		**Mino * PT intervention**	0.35	0.84
		**LM11A-31 * PT intervention**	0.65	0.72
	**Type 1 error head tilts**	**Mino**	1.48	0.22
		**LM11A-31**	3.23	0.07
		**PT intervention**	24.40	0.00
		**Mino * LM11A-31**	1.48	0.22
		**Mino * PT intervention**	7.45	0.02
		**LM11A-31 * PT intervention**	15.35	0.00
	**Type 2 error total**	**Mino**	59.23	0.00
		**LM11A-31**	0.67	0.41
		**PT intervention**	2.37	0.31
		**Mino * LM11A-31**	0.17	0.68
		**Mino * PT intervention**	11.16	0.00
		**LM11A-31 * PT intervention**	1.56	0.46
	**Type 2 error switch hands**	**Mino**	2.76	0.10
		**LM11A-31**	0.11	0.75
		**PT intervention**	3.71	0.16
		**Mino * LM11A-31**	0.72	0.40
		**Mino * PT intervention**	3.79	0.15
		**LM11A-31 * PT intervention**	0.04	0.98
	**Type 2 error uses mouth**	**Mino**	42.14	0.00
		**LM11A-31**	0.21	0.65
		**PT intervention**	0.25	0.89
		**Mino * LM11A-31**	1.55	0.21
		**Mino * PT intervention**	12.08	0.00
		**LM11A-31 * PT intervention**	13.62	0.00
**Morris water maze**	**Visible whole pool latency**	**Mino**	0.03	0.86
		**LM11A-31**	0.74	0.39
		**PT intervention**	4.83	0.01
		**Mino * LM11A-31**	0.18	0.67
		**Mino * PT intervention**	0.53	0.59
		**LM11A-31 * PT intervention**	0.81	0.45
	**Visible whole pool distance**	**Mino**	0.30	0.58
		**LM11A-31**	1.52	0.22

		PT intervention	8.29	0.00
		Mino * LM11A-31	0.26	0.61
		Mino * PT intervention	0.65	0.53
		LM11A-31 * PT intervention	0.49	0.62
	Visible thigmotaxic area latency	Mino	0.83	0.36
		LM11A-31	0.20	0.66
		PT intervention	4.63	0.01
		Mino * LM11A-31	0.01	0.92
		Mino * PT intervention	0.34	0.71
		LM11A-31 * PT intervention	0.21	0.81
	Visible thigmotaxic area distance	Mino	0.06	0.81
		LM11A-31	0.11	0.74
		PT intervention	9.53	0.00
		Mino * LM11A-31	0.04	0.85
		Mino * PT intervention	0.33	0.72
		LM11A-31 * PT intervention	1.51	0.22
	Hidden whole pool latency	Mino	5.02	0.03
		LM11A-31	18.43	0.00
		PT intervention	1.35	0.26
		Mino * LM11A-31	8.79	0.00
		Mino * PT intervention	3.83	0.02
		LM11A-31 * PT intervention	12.63	0.00
	Hidden whole pool distance	Mino	4.62	0.03
		LM11A-31	15.25	0.00
		PT intervention	1.02	0.36
		Mino * LM11A-31	4.14	0.04
		Mino * PT intervention	2.41	0.10
		LM11A-31 * PT intervention	11.13	0.00
	Hidden thigmotaxic area latency	Mino	1.07	0.30
		LM11A-31	8.17	0.01
		PT intervention	3.06	0.05
		Mino * LM11A-31	12.21	0.00
		Mino * PT intervention	0.31	0.73
		LM11A-31 * PT intervention	9.84	0.00
	Hidden thigmotaxic area distance	Mino	0.70	0.41
		LM11A-31	4.84	0.03
		PT intervention	2.15	0.12
		Mino * LM11A-31	6.20	0.01
		Mino * PT intervention	0.06	0.94
		LM11A-31 * PT intervention	8.82	0.00
	Hidden whole pool - thigmotaxic area latency	Mino	3.62	0.06
		LM11A-31	8.83	0.00
		PT intervention	3.87	0.02
		Mino * LM11A-31	0.78	0.38
		Mino * PT intervention	4.08	0.02
		LM11A-31 * PT intervention	7.81	0.00
	Hidden whole pool - thigmotaxic area distance	Mino	4.44	0.04
		LM11A-31	11.81	0.00
		PT intervention	3.80	0.03
		Mino * LM11A-31	1.03	0.31
		Mino * PT intervention	3.21	0.04
		LM11A-31 * PT intervention	7.54	0.00
	Probe thigmotaxic area latency	Mino	12.19	0.00
		LM11A-31	0.83	0.37
		PT intervention	7.08	0.00
		Mino * LM11A-31	2.37	0.13
		Mino * PT intervention	2.02	0.14
		LM11A-31 * PT intervention	3.18	0.05

	**Probe thigmotaxic area distance**	**Mino**	10.50	0.00
		**LM11A-31**	0.08	0.78
		**PT intervention**	4.41	0.01
		**Mino * LM11A-31**	1.04	0.31
		**Mino * PT intervention**	2.48	0.09
		**LM11A-31 * PT intervention**	3.82	0.03
	**Probe large platform frequency**	**Mino**	0.07	0.79
		**LM11A-31**	10.45	0.00
		**PT intervention**	3.50	0.03
		**Mino * LM11A-31**	3.46	0.07
		**Mino * PT intervention**	0.31	0.73
		**LM11A-31 * PT intervention**	4.92	0.01
	**Probe large platform 1st latency**	**Mino**	0.00	0.99
		**LM11A-31**	2.81	0.10
		**PT intervention**	0.66	0.52
		**Mino * LM11A-31**	1.81	0.18
		**Mino * PT intervention**	0.74	0.48
		**LM11A-31 * PT intervention**	4.01	0.02
	**Probe platform frequency**	**Mino**	0.81	0.37
		**LM11A-31**	8.12	0.01
		**PT intervention**	2.31	0.10
		**Mino * LM11A-31**	7.42	0.01
		**Mino * PT intervention**	1.62	0.20
		**LM11A-31 * PT intervention**	6.27	0.00
	**Probe target quadrant duration**	**Mino**	0.09	0.76
		**LM11A-31**	11.54	0.00
		**PT intervention**	12.22	0.00
		**Mino * LM11A-31**	0.93	0.34
		**Mino * PT intervention**	0.12	0.89
		**LM11A-31 * PT intervention**	4.75	0.01
	**Probe target quadrant 1st latency**	**Mino**	0.43	0.52
		**LM11A-31**	0.01	0.91
		**PT intervention**	2.20	0.12
		**Mino * LM11A-31**	0.01	0.92
		**Mino * PT intervention**	1.64	0.20
		**LM11A-31 * PT intervention**	1.78	0.17
	**Probe target quadrant % duration**	**Mino**	0.11	0.74
		**LM11A-31**	11.41	0.00
		**PT intervention**	11.70	0.00
		**Mino * LM11A-31**	1.14	0.29
		**Mino * PT intervention**	0.14	0.87
		**LM11A-31 * PT intervention**	4.75	0.01

PT intervention- physical therapy intervention (i.e., no physical therapy, physical therapy alone and physical therapy in combination with botox).

Supplementary Table 3: Univariate effects – Double-combo LM11A31 and Triple-combo experiments

		Effect	F or Wald Chi-Square	Sig.
	**Weight Day 0**	**Mino**	0.07	0.79
		**LM11A-31**	1.55	0.22
		**PT intervention**	49.82	0.00
		**Mino * LM11A-31**	0.26	0.61
		**LM11A-31 * PT intervention**	1.32	0.27
	**Brain lesion %**	**Mino**	1.68	0.20
		**LM11A-31**	1.07	0.30
		**PT intervention**	0.55	0.58
		**Mino * LM11A-31**	0.01	0.92
		**LM11A-31 * PT intervention**	0.04	0.96
	**DCX Ipsilateral**	**Mino**	0.65	0.42
		**LM11A-31**	0.18	0.67
		**PT intervention**	30.88	0.00
		**Mino * LM11A-31**	0.26	0.61
		**LM11A-31 * PT intervention**	0.55	0.58
	**DCX Contralateral**	**Mino**	1.62	0.21
		**LM11A-31**	0.22	0.64
		**PT intervention**	56.20	0.00
		**Mino * LM11A-31**	2.81	0.10
		**LM11A-31 * PT intervention**	1.35	0.27
	**Ki67 Ipsilateral**	**Mino**	0.04	0.84
		**LM11A-31**	0.27	0.61
		**PT intervention**	50.14	0.00
		**Mino * LM11A-31**	0.11	0.75
		**LM11A-31 * PT intervention**	0.12	0.89
	**Ki67 Contralateral**	**Mino**	3.23	0.08
		**LM11A-31**	5.58	0.02
		**PT intervention**	185.19	0.00
		**Mino * LM11A-31**	12.31	0.00
		**LM11A-31 * PT intervention**	2.68	0.08
**SLT**	**Time to remove right paw**	**Mino**	0.26	0.61
		**LM11A-31**	0.49	0.49
		**PT intervention**	6.41	0.00
		**Mino * LM11A-31**	3.58	0.06
		**LM11A-31 * PT intervention**	2.12	0.13
	**Cylinder % right paw**	**Mino**	0.00	0.99
		**LM11A-31**	0.08	0.78
		**PT intervention**	8.48	0.00
		**Mino * LM11A-31**	0.05	0.82
		**LM11A-31 * PT intervention**	0.15	0.86
**Vermicelli handling test**	**Type 1 error total**	**Mino**	0.00	0.96
		**LM11A-31**	0.33	0.56
		**PT intervention**	1.47	0.48
		**Mino * LM11A-31**	0.05	0.83
		**LM11A-31 * PT intervention**	3.49	0.18
	**Type 1 error hands apart**	**Mino**	0.18	0.68
		**LM11A-31**	0.44	0.51
		**PT intervention**	0.88	0.64
		**Mino * LM11A-31**	0.00	0.95
		**LM11A-31 * PT intervention**	1.78	0.41

	**Type 2 error total**	**Mino**	23.16	0.00
		**LM11A-31**	6.32	0.01
		**PT intervention**	12.12	0.00
		**Mino * LM11A-31**	0.17	0.68
		**LM11A-31 * PT intervention**	6.81	0.03
	**Type 2 error switch hands**	**Mino**	0.42	0.52
		**LM11A-31**	1.20	0.27
		**PT intervention**	1.05	0.59
		**Mino * LM11A-31**	0.72	0.40
		**LM11A-31 * PT intervention**	1.56	0.46
	**Type 2 error uses mouth**	**Mino**	12.04	0.00
		**LM11A-31**	2.44	0.12
		**PT intervention**	20.34	0.00
		**Mino * LM11A-31**	1.55	0.21
		**LM11A-31 * PT intervention**	19.32	0.00
	**Type 2 error drop/ flips pasta**	**Mino**	5.71	0.02
		**LM11A-31**	1.65	0.20
		**PT intervention**	1.06	0.59
		**Mino * LM11A-31**	0.00	0.99
		**LM11A-31 * PT intervention**	14.71	0.00
**Morris water maze**	**Visible whole pool latency**	**Mino**	0.78	0.38
		**LM11A-31**	0.00	0.99
		**PT intervention**	5.47	0.01
		**Mino * LM11A-31**	0.19	0.67
		**LM11A-31 * PT intervention**	0.54	0.58
	**Visible whole pool distance**	**Mino**	0.26	0.61
		**LM11A-31**	0.02	0.90
		**PT intervention**	11.50	0.00
		**Mino * LM11A-31**	0.32	0.57
		**LM11A-31 * PT intervention**	0.01	0.99
	**Visible thigmotaxic area latency**	**Mino**	0.04	0.85
		**LM11A-31**	0.42	0.52
		**PT intervention**	4.46	0.01
		**Mino * LM11A-31**	0.01	0.92
		**LM11A-31 * PT intervention**	0.03	0.97
	**Visible thigmotaxic area distance**	**Mino**	0.17	0.69
		**LM11A-31**	0.30	0.58
		**PT intervention**	9.90	0.00
		**Mino * LM11A-31**	0.04	0.85
		**LM11A-31 * PT intervention**	0.44	0.65
	**Visible whole pool - thigmotaxic area latency**	**Mino**	1.82	0.18
		**LM11A-31**	1.22	0.27
		**PT intervention**	4.83	0.01
		**Mino * LM11A-31**	0.42	0.52
		**LM11A-31 * PT intervention**	1.72	0.19
	**Visible whole pool - thigmotaxic area distance**	**Mino**	3.11	0.08
		**LM11A-31**	0.49	0.49
		**PT intervention**	5.92	0.00
		**Mino * LM11A-31**	0.69	0.41
		**LM11A-31 * PT intervention**	1.79	0.17
	**Hidden whole pool latency**	**Mino**	0.16	0.69
		**LM11A-31**	10.47	0.00
		**PT intervention**	3.20	0.05
		**Mino * LM11A-31**	13.87	0.00
		**LM11A-31 * PT intervention**	10.11	0.00

	**Hidden whole pool distance**	**Mino**	0.22	0.64
		**LM11A-31**	7.07	0.01
		**PT intervention**	0.67	0.51
		**Mino * LM11A-31**	8.09	0.01
		**LM11A-31 * PT intervention**	10.80	0.00
	**Hidden thigmotaxic area latency**	**Mino**	0.04	0.85
		**LM11A-31**	10.19	0.00
		**PT intervention**	1.94	0.15
		**Mino * LM11A-31**	16.09	0.00
		**LM11A-31 * PT intervention**	10.39	0.00
	**Hidden thigmotaxic area distance**	**Mino**	0.23	0.63
		**LM11A-31**	6.26	0.01
		**PT intervention**	1.97	0.15
		**Mino * LM11A-31**	7.41	0.01
		**LM11A-31 * PT intervention**	8.02	0.00
	**Hidden whole pool - thigmotaxic area latency**	**Mino**	0.15	0.70
		**LM11A-31**	2.07	0.15
		**PT intervention**	1.39	0.25
		**Mino * LM11A-31**	1.74	0.19
		**LM11A-31 * PT intervention**	2.28	0.11
	**Hidden whole pool - thigmotaxic area distance**	**Mino**	0.08	0.79
		**LM11A-31**	3.22	0.08
		**PT intervention**	0.20	0.82
		**Mino * LM11A-31**	3.50	0.07
		**LM11A-31 * PT intervention**	6.11	0.00
	**Probe whole pool distance**	**Mino**	0.07	0.79
		**LM11A-31**	0.00	0.99
		**PT intervention**	1.96	0.15
		**Mino * LM11A-31**	0.08	0.78
		**LM11A-31 * PT intervention**	2.39	0.10
	**Probe thigmotaxic area latency**	**Mino**	3.50	0.07
		**LM11A-31**	0.37	0.54
		**PT intervention**	2.10	0.13
		**Mino * LM11A-31**	2.25	0.14
		**LM11A-31 * PT intervention**	3.49	0.04
	**Probe thigmotaxic area distance**	**Mino**	1.67	0.20
		**LM11A-31**	0.00	0.99
		**PT intervention**	0.11	0.90
		**Mino * LM11A-31**	0.99	0.32
		**LM11A-31 * PT intervention**	5.35	0.01
	**Probe large platform frequency**	**Mino**	0.00	0.95
		**LM11A-31**	3.81	0.05
		**PT intervention**	8.71	0.00
		**Mino * LM11A-31**	3.37	0.07
		**LM11A-31 * PT intervention**	2.72	0.07
	**Probe large platform latency 1st latency**	**Mino**	2.09	**0.15**
		**LM11A-31**	1.73	**0.19**
		**PT intervention**	0.50	**0.61**
		**Mino * LM11A-31**	1.92	**0.17**
		**LM11A-31 * PT intervention**	3.02	**0.05**
	**Probe platform frequency**	**Mino**	0.07	0.80
		**LM11A-31**	4.24	0.04
		**PT intervention**	0.67	0.52
		**Mino * LM11A-31**	6.49	0.01
		**LM11A-31 * PT intervention**	4.73	0.01

	**Probe target quadrant duration**	**Mino**	0.00	0.98
		**LM11A-31**	6.06	0.02
		**PT intervention**	24.73	0.00
		**Mino * LM11A-31**	1.12	0.29
		**LM11A-31 * PT intervention**	4.29	0.02
	**Probe target quadrant 1st latency**	**Mino**	0.72	0.40
		**LM11A-31**	0.00	0.96
		**PT intervention**	3.19	0.05
		**Mino * LM11A-31**	0.01	0.92
		**LM11A-31 * PT intervention**	1.61	0.21
	**Probe target quadrant % duration**	**Mino**	0.03	0.87
		**LM11A-31**	6.34	0.01
		**PT intervention**	24.34	0.00
		**Mino * LM11A-31**	1.36	0.25
		**LM11A-31 * PT intervention**	4.22	0.02

PT intervention- physical therapy intervention (i.e., no physical therapy, physical therapy alone and physical therapy in combination with botox).

Supplementary Table 4: Hypothesis testing of 3-dimensional PCA solution – Double-combo LM11A31 and Triple-combo experiments

		df			
	**Effect**	**Numerator**	**Denominator**	**F**	**Sig.**
**PC1**	**Mino**	1	85	0.24	0.26
	**LM11A-31**	1	85	5.71	0.02
	**PT intervention**	2	85	3.06	0.05
	**Mino * LM11A-31**	1	85	5.30	0.02
	**LM11A-31 * PT intervention**	2	85	6.75	0.00
**PC2**	**Mino**	1	85	0.84	0.36
	**LM11A-31**	1	85	12.14	0.00
	**PT intervention**	2	85	35.10	0.00
	**Mino * LM11A-31**	1	85	17.06	0.00
	**LM11A-31 * PT intervention**	2	85	12.82	0.00

PT intervention- physical therapy intervention (i.e., no physical therapy, physical therapy alone and physical therapy in combination with botox).
Estimated marginal means and error terms are plotted in Fig. 2 e-k.

Supplementary Table 5: Hypothesis testing of 2-dimensional PCA solution – all experiments combined

		df			
	**Effect**	**Numerator**	**Denominator**	**F**	**Sig.**
**PC1**	**Mino**	1	113	2.41	0.12
	**LM11A-31**	1	113	14.20	0.00
	**PT intervention**	2	113	1.51	0.23
	**Mino * LM11A-31**	1	113	3.42	0.07
	**Mino * PT intervention**	2	113	0.73	0.48
	**LM11A-31 * PT intervention**	2	113	9.99	0.00

PT intervention- physical therapy intervention (i.e., no physical therapy, physical therapy alone and physical therapy in combination with botox).

Estimated marginal means and error terms are plotted in Supplementary Fig. 5 e-f.

SPSS Syntax for 6-dimensional NL-PCA: Double-combo LM11A-31 and Triple-combo experiments (Supplementary Fig. 3b)

```
CATPCA VARIABLES=
Weight_Day_0
Weight_Day_10
BrainLesion_percentage
DCXIPSI
DCXContra
Ki67IPSI
Ki67Contra
SLT_Time2Remove_Leftpaw_Avg
SLT_Time2Remove_Rightpaw_Avg
Cylinder_Leftpaw_perc
Cylinder_Rightpaw_perc
Pastatest_Type1_Total_sum
Pastatest_Type1_handsapart_sum
Pastatest_Type1_hunching_sum
Pastatest_Type1_headtilts_sum
Pastatest_Type2_Total_sum
Pastatest_Type2_Switchhands_sum
Pastatest_Type2_usesmouth_sum
Pastatest_Type2_breakspasta_sum
Pastatest_Type2_dropflip_sum
WP_Visible_Latency_AVG
WP_Visible_Distance_AVG
TM_Visible_Latency_AVG
TM_Visible_Distance_AVG
WPTM_Visible_Latency_AVG
WPTM_Visible_Distance_AVG
WP_Hidden_Latency_Day_AVG
WP_Hidden_Distance_Day_AVG
TM_Hidden_Latency_Day_AVG
TM_Hidden_Distance_Day_AVG
WPTM_Hidden_Latency_Day_AVG
WPTM_Hidden_Distance_Day_AVG
WP_Probe_Distance
TM_Probe_Latency
TM_Probe_Distance
WPTM_Probe_Distance
LP_Probe_Frequency
LP_Probe_Latency_FirstOcc
P_Probe_Frequency
P_Probe_Latency_FirstOcc
```

```
TQ_Probe_Duration
TQ_Probe_Latency_FirstOcc
TQ_Probe_PercTime
  /ANALYSIS=
Weight_Day_0(WEIGHT=1,LEVEL= NUME)
Weight_Day_10(WEIGHT=1,LEVEL= NUME)
BrainLesion_percentage(WEIGHT=1,LEVEL= NUME)
DCXIPSI(WEIGHT=1,LEVEL= NUME)
DCXContra(WEIGHT=1,LEVEL= NUME)
Ki67IPSI(WEIGHT=1,LEVEL= NUME)
Ki67Contra(WEIGHT=1,LEVEL= NUME)
SLT_Time2Remove_Leftpaw_Avg(WEIGHT=1,LEVEL= NUME)
SLT_Time2Remove_Rightpaw_Avg(WEIGHT=1,LEVEL= NUME)
Cylinder_Leftpaw_perc(WEIGHT=1,LEVEL= NUME)
Cylinder_Rightpaw_perc(WEIGHT=1,LEVEL= NUME)
Pastatest_Type1_Total_sum(WEIGHT=1,LEVEL= ORDI)
Pastatest_Type1_handsapart_sum(WEIGHT=1,LEVEL= ORDI)
Pastatest_Type1_hunching_sum(WEIGHT=1,LEVEL= ORDI)
Pastatest_Type1_headtilts_sum(WEIGHT=1,LEVEL= ORDI)
Pastatest_Type2_Total_sum(WEIGHT=1,LEVEL= ORDI)
Pastatest_Type2_Switchhands_sum(WEIGHT=1,LEVEL= ORDI)
Pastatest_Type2_usesmouth_sum(WEIGHT=1,LEVEL= ORDI)
Pastatest_Type2_breakspasta_sum(WEIGHT=1,LEVEL= ORDI)
Pastatest_Type2_dropflip_sum(WEIGHT=1,LEVEL= ORDI)
WP_Visible_Latency_AVG(WEIGHT=1,LEVEL= NUME)
WP_Visible_Distance_AVG(WEIGHT=1,LEVEL= NUME)
TM_Visible_Latency_AVG(WEIGHT=1,LEVEL= NUME)
TM_Visible_Distance_AVG(WEIGHT=1,LEVEL= NUME)
WPTM_Visible_Latency_AVG(WEIGHT=1,LEVEL= NUME)
WPTM_Visible_Distance_AVG(WEIGHT=1,LEVEL= NUME)
WP_Hidden_Latency_Day_AVG(WEIGHT=1,LEVEL= NUME)
WP_Hidden_Distance_Day_AVG(WEIGHT=1,LEVEL= NUME)
TM_Hidden_Latency_Day_AVG(WEIGHT=1,LEVEL= NUME)
TM_Hidden_Distance_Day_AVG(WEIGHT=1,LEVEL= NUME)
WPTM_Hidden_Latency_Day_AVG(WEIGHT=1,LEVEL= NUME)
WPTM_Hidden_Distance_Day_AVG(WEIGHT=1,LEVEL= NUME)
WP_Probe_Distance(WEIGHT=1,LEVEL= NUME)
TM_Probe_Latency(WEIGHT=1,LEVEL= NUME)
TM_Probe_Distance(WEIGHT=1,LEVEL= NUME)
WPTM_Probe_Distance(WEIGHT=1,LEVEL= NUME)
LP_Probe_Frequency(WEIGHT=1,LEVEL= NUME)
LP_Probe_Latency_FirstOcc(WEIGHT=1,LEVEL= NUME)
P_Probe_Frequency(WEIGHT=1,LEVEL= NUME)
P_Probe_Latency_FirstOcc(WEIGHT=1,LEVEL= NUME)
```

TQ_Probe_Duration(WEIGHT=1,LEVEL= NUME)
TQ_Probe_Latency_FirstOcc(WEIGHT=1,LEVEL= NUME)
TQ_Probe_PercTime(WEIGHT=1,LEVEL= NUME)
/DISCRETIZATION=
Weight_Day_0(MULTIPLYING)
Weight_Day_10(MULTIPLYING)
BrainLesion_percentage(MULTIPLYING)
DCXIPSI(MULTIPLYING)
DCXContra(MULTIPLYING)
Ki67IPSI(MULTIPLYING)
Ki67Contra(MULTIPLYING)
SLT_Time2Remove_Leftpaw_Avg(MULTIPLYING)
SLT_Time2Remove_Rightpaw_Avg(MULTIPLYING)
Cylinder_Leftpaw_perc(MULTIPLYING)
Cylinder_Rightpaw_perc(MULTIPLYING)
Pastatest_Type1_Total_sum(Ranking)
Pastatest_Type1_handsapart_sum(Ranking)
Pastatest_Type1_hunching_sum(Ranking)
Pastatest_Type1_headtilts_sum(Ranking)
Pastatest_Type2_Total_sum(Ranking)
Pastatest_Type2_Switchhands_sum(Ranking)
Pastatest_Type2_usesmouth_sum(Ranking)
Pastatest_Type2_breakspasta_sum(Ranking)
Pastatest_Type2_dropflip_sum(Ranking)
WP_Visible_Latency_AVG(MULTIPLYING)
WP_Visible_Distance_AVG(MULTIPLYING)
TM_Visible_Latency_AVG(MULTIPLYING)
TM_Visible_Distance_AVG(MULTIPLYING)
WPTM_Visible_Latency_AVG(MULTIPLYING)
WPTM_Visible_Distance_AVG(MULTIPLYING)
WP_Hidden_Latency_Day_AVG(MULTIPLYING)
WP_Hidden_Distance_Day_AVG(MULTIPLYING)
TM_Hidden_Latency_Day_AVG(MULTIPLYING)
TM_Hidden_Distance_Day_AVG(MULTIPLYING)
WPTM_Hidden_Latency_Day_AVG(MULTIPLYING)
WPTM_Hidden_Distance_Day_AVG(MULTIPLYING)
WP_Probe_Distance(MULTIPLYING)
TM_Probe_Latency(MULTIPLYING)
TM_Probe_Distance(MULTIPLYING)
WPTM_Probe_Distance(MULTIPLYING)
LP_Probe_Frequency(MULTIPLYING)
LP_Probe_Latency_FirstOcc(MULTIPLYING)
P_Probe_Frequency(MULTIPLYING)
P_Probe_Latency_FirstOcc(MULTIPLYING)

TQ_Probe_Duration(MULTIPLYING)
TQ_Probe_Latency_FirstOcc(MULTIPLYING)
TQ_Probe_PercTime(MULTIPLYING)
/MISSING=
Weight_Day_0(ACTIVE,MODEIMPU)
Weight_Day_10(ACTIVE,MODEIMPU)
BrainLesion_percentage(ACTIVE,MODEIMPU)
DCXIPSI(ACTIVE,MODEIMPU)
DCXContra(ACTIVE,MODEIMPU)
Ki67IPSI(ACTIVE,MODEIMPU)
Ki67Contra(ACTIVE,MODEIMPU)
SLT_Time2Remove_Leftpaw_Avg(ACTIVE,MODEIMPU)
SLT_Time2Remove_Rightpaw_Avg(ACTIVE,MODEIMPU)
Cylinder_Leftpaw_perc(ACTIVE,MODEIMPU)
Cylinder_Rightpaw_perc(ACTIVE,MODEIMPU)
Pastatest_Type1_Total_sum(ACTIVE,MODEIMPU)
Pastatest_Type1_handsapart_sum(ACTIVE,MODEIMPU)
Pastatest_Type1_hunching_sum(ACTIVE,MODEIMPU)
Pastatest_Type1_headtilts_sum(ACTIVE,MODEIMPU)
Pastatest_Type2_Total_sum(ACTIVE,MODEIMPU)
Pastatest_Type2_Switchhands_sum(ACTIVE,MODEIMPU)
Pastatest_Type2_usesmouth_sum(ACTIVE,MODEIMPU)
Pastatest_Type2_breakspasta_sum(ACTIVE,MODEIMPU)
Pastatest_Type2_dropflip_sum(ACTIVE,MODEIMPU)
WP_Visible_Latency_AVG(ACTIVE,MODEIMPU)
WP_Visible_Distance_AVG(ACTIVE,MODEIMPU)
TM_Visible_Latency_AVG(ACTIVE,MODEIMPU)
TM_Visible_Distance_AVG(ACTIVE,MODEIMPU)
WPTM_Visible_Latency_AVG(ACTIVE,MODEIMPU)
WPTM_Visible_Distance_AVG(ACTIVE,MODEIMPU)
WP_Hidden_Latency_Day_AVG(ACTIVE,MODEIMPU)
WP_Hidden_Distance_Day_AVG(ACTIVE,MODEIMPU)
TM_Hidden_Latency_Day_AVG(ACTIVE,MODEIMPU)
TM_Hidden_Distance_Day_AVG(ACTIVE,MODEIMPU)
WPTM_Hidden_Latency_Day_AVG(ACTIVE,MODEIMPU)
WPTM_Hidden_Distance_Day_AVG(ACTIVE,MODEIMPU)
WP_Probe_Distance(ACTIVE,MODEIMPU)
TM_Probe_Latency(ACTIVE,MODEIMPU)
TM_Probe_Distance(ACTIVE,MODEIMPU)
WPTM_Probe_Distance(ACTIVE,MODEIMPU)
LP_Probe_Frequency(ACTIVE,MODEIMPU)
LP_Probe_Latency_FirstOcc(ACTIVE,MODEIMPU)
P_Probe_Frequency(ACTIVE,MODEIMPU)
P_Probe_Latency_FirstOcc(ACTIVE,MODEIMPU)

```
TQ_Probe_Duration(ACTIVE,MODEIMPU)
TQ_Probe_Latency_FirstOcc(ACTIVE,MODEIMPU)
TQ_Probe_PercTime(ACTIVE,MODEIMPU)
  /DIMENSION=6
  /NORMALIZATION=VPRINCIPAL
  /MAXITER=100
  /CRITITER=.00001
/RESAMPLE=BOOTSTRAP (2000 95 BALANCED PROCRU)
  /PRINT DESCRIP VAF LOADING CORR OCORR OBJECT
  /PLOT=BIPLOT(LOADING) (20) OBJECT (20)
/SAVE=OBJECT.
```

SPSS Syntax for 3-dimensional NL-PCA: Double-combo LM11A-31 and Triple-combo experiments (Figure 2 and Supplementary Fig. 4a)

```
CATPCA VARIABLES=
Weight_Day_0
Weight_Day_10
BrainLesion_percentage
DCXIPSI
DCXContra
Ki67IPSI
Ki67Contra
SLT_Time2Remove_Leftpaw_Avg
SLT_Time2Remove_Rightpaw_Avg
Cylinder_Leftpaw_perc
Cylinder_Rightpaw_perc
Pastatest_Type1_Total_sum
Pastatest_Type1_handsapart_sum
Pastatest_Type1_hunching_sum
Pastatest_Type1_headtilts_sum
Pastatest_Type2_Total_sum
Pastatest_Type2_Switchhands_sum
Pastatest_Type2_usesmouth_sum
Pastatest_Type2_breakspasta_sum
Pastatest_Type2_dropflip_sum
WP_Visible_Latency_AVG
WP_Visible_Distance_AVG
TM_Visible_Latency_AVG
TM_Visible_Distance_AVG
WPTM_Visible_Latency_AVG
WPTM_Visible_Distance_AVG
WP_Hidden_Latency_Day_AVG
WP_Hidden_Distance_Day_AVG
```

TM_Hidden_Latency_Day_AVG
TM_Hidden_Distance_Day_AVG
WPTM_Hidden_Latency_Day_AVG
WPTM_Hidden_Distance_Day_AVG
WP_Probe_Distance
TM_Probe_Latency
TM_Probe_Distance
WPTM_Probe_Distance
LP_Probe_Frequency
LP_Probe_Latency_FirstOcc
P_Probe_Frequency
P_Probe_Latency_FirstOcc
TQ_Probe_Duration
TQ_Probe_Latency_FirstOcc
TQ_Probe_PercTime
/ANALYSIS=
Weight_Day_0(WEIGHT=1,LEVEL= NUME)
Weight_Day_10(WEIGHT=1,LEVEL= NUME)
BrainLesion_percentage(WEIGHT=1,LEVEL= NUME)
DCXIPSI(WEIGHT=1,LEVEL= NUME)
DCXContra(WEIGHT=1,LEVEL= NUME)
Ki67IPSI(WEIGHT=1,LEVEL= NUME)
Ki67Contra(WEIGHT=1,LEVEL= NUME)
SLT_Time2Remove_Leftpaw_Avg(WEIGHT=1,LEVEL= NUME)
SLT_Time2Remove_Rightpaw_Avg(WEIGHT=1,LEVEL= NUME)
Cylinder_Leftpaw_perc(WEIGHT=1,LEVEL= NUME)
Cylinder_Rightpaw_perc(WEIGHT=1,LEVEL= NUME)
Pastatest_Type1_Total_sum(WEIGHT=1,LEVEL= ORDI)
Pastatest_Type1_handsapart_sum(WEIGHT=1,LEVEL= ORDI)
Pastatest_Type1_hunching_sum(WEIGHT=1,LEVEL= ORDI)
Pastatest_Type1_headtilts_sum(WEIGHT=1,LEVEL= ORDI)
Pastatest_Type2_Total_sum(WEIGHT=1,LEVEL= ORDI)
Pastatest_Type2_Switchhands_sum(WEIGHT=1,LEVEL= ORDI)
Pastatest_Type2_usesmouth_sum(WEIGHT=1,LEVEL= ORDI)
Pastatest_Type2_breakspasta_sum(WEIGHT=1,LEVEL= ORDI)
Pastatest_Type2_dropflip_sum(WEIGHT=1,LEVEL= ORDI)
WP_Visible_Latency_AVG(WEIGHT=1,LEVEL= NUME)
WP_Visible_Distance_AVG(WEIGHT=1,LEVEL= NUME)
TM_Visible_Latency_AVG(WEIGHT=1,LEVEL= NUME)
TM_Visible_Distance_AVG(WEIGHT=1,LEVEL= NUME)
WPTM_Visible_Latency_AVG(WEIGHT=1,LEVEL= NUME)
WPTM_Visible_Distance_AVG(WEIGHT=1,LEVEL= NUME)
WP_Hidden_Latency_Day_AVG(WEIGHT=1,LEVEL= NUME)
WP_Hidden_Distance_Day_AVG(WEIGHT=1,LEVEL= NUME)

TM_Hidden_Latency_Day_AVG(WEIGHT=1,LEVEL= NUME)
TM_Hidden_Distance_Day_AVG(WEIGHT=1,LEVEL= NUME)
WPTM_Hidden_Latency_Day_AVG(WEIGHT=1,LEVEL= NUME)
WPTM_Hidden_Distance_Day_AVG(WEIGHT=1,LEVEL= NUME)
WP_Probe_Distance(WEIGHT=1,LEVEL= NUME)
TM_Probe_Latency(WEIGHT=1,LEVEL= NUME)
TM_Probe_Distance(WEIGHT=1,LEVEL= NUME)
WPTM_Probe_Distance(WEIGHT=1,LEVEL= NUME)
LP_Probe_Frequency(WEIGHT=1,LEVEL= NUME)
LP_Probe_Latency_FirstOcc(WEIGHT=1,LEVEL= NUME)
P_Probe_Frequency(WEIGHT=1,LEVEL= NUME)
P_Probe_Latency_FirstOcc(WEIGHT=1,LEVEL= NUME)
TQ_Probe_Duration(WEIGHT=1,LEVEL= NUME)
TQ_Probe_Latency_FirstOcc(WEIGHT=1,LEVEL= NUME)
TQ_Probe_PercTime(WEIGHT=1,LEVEL= NUME)
/DISCRETIZATION=
Weight_Day_0(MULTIPLYING)
Weight_Day_10(MULTIPLYING)
BrainLesion_percentage(MULTIPLYING)
DCXIPSI(MULTIPLYING)
DCXContra(MULTIPLYING)
Ki67IPSI(MULTIPLYING)
Ki67Contra(MULTIPLYING)
SLT_Time2Remove_Leftpaw_Avg(MULTIPLYING)
SLT_Time2Remove_Rightpaw_Avg(MULTIPLYING)
Cylinder_Leftpaw_perc(MULTIPLYING)
Cylinder_Rightpaw_perc(MULTIPLYING)
Pastatest_Type1_Total_sum(Ranking)
Pastatest_Type1_handsapart_sum(Ranking)
Pastatest_Type1_hunching_sum(Ranking)
Pastatest_Type1_headtilts_sum(Ranking)
Pastatest_Type2_Total_sum(Ranking)
Pastatest_Type2_Switchhands_sum(Ranking)
Pastatest_Type2_usesmouth_sum(Ranking)
Pastatest_Type2_breakspasta_sum(Ranking)
Pastatest_Type2_dropflip_sum(Ranking)
WP_Visible_Latency_AVG(MULTIPLYING)
WP_Visible_Distance_AVG(MULTIPLYING)
TM_Visible_Latency_AVG(MULTIPLYING)
TM_Visible_Distance_AVG(MULTIPLYING)
WPTM_Visible_Latency_AVG(MULTIPLYING)
WPTM_Visible_Distance_AVG(MULTIPLYING)
WP_Hidden_Latency_Day_AVG(MULTIPLYING)
WP_Hidden_Distance_Day_AVG(MULTIPLYING)

TM_Hidden_Latency_Day_AVG(MULTIPLYING)
TM_Hidden_Distance_Day_AVG(MULTIPLYING)
WPTM_Hidden_Latency_Day_AVG(MULTIPLYING)
WPTM_Hidden_Distance_Day_AVG(MULTIPLYING)
WP_Probe_Distance(MULTIPLYING)
TM_Probe_Latency(MULTIPLYING)
TM_Probe_Distance(MULTIPLYING)
WPTM_Probe_Distance(MULTIPLYING)
LP_Probe_Frequency(MULTIPLYING)
LP_Probe_Latency_FirstOcc(MULTIPLYING)
P_Probe_Frequency(MULTIPLYING)
P_Probe_Latency_FirstOcc(MULTIPLYING)
TQ_Probe_Duration(MULTIPLYING)
TQ_Probe_Latency_FirstOcc(MULTIPLYING)
TQ_Probe_PercTime(MULTIPLYING)
/MISSING=
Weight_Day_0(ACTIVE,MODEIMPU)
Weight_Day_10(ACTIVE,MODEIMPU)
BrainLesion_percentage(ACTIVE,MODEIMPU)
DCXIPSI(ACTIVE,MODEIMPU)
DCXContra(ACTIVE,MODEIMPU)
Ki67IPSI(ACTIVE,MODEIMPU)
Ki67Contra(ACTIVE,MODEIMPU)
SLT_Time2Remove_Leftpaw_Avg(ACTIVE,MODEIMPU)
SLT_Time2Remove_Rightpaw_Avg(ACTIVE,MODEIMPU)
Cylinder_Leftpaw_perc(ACTIVE,MODEIMPU)
Cylinder_Rightpaw_perc(ACTIVE,MODEIMPU)
Pastatest_Type1_Total_sum(ACTIVE,MODEIMPU)
Pastatest_Type1_handsapart_sum(ACTIVE,MODEIMPU)
Pastatest_Type1_hunching_sum(ACTIVE,MODEIMPU)
Pastatest_Type1_headtilts_sum(ACTIVE,MODEIMPU)
Pastatest_Type2_Total_sum(ACTIVE,MODEIMPU)
Pastatest_Type2_Switchhands_sum(ACTIVE,MODEIMPU)
Pastatest_Type2_usesmouth_sum(ACTIVE,MODEIMPU)
Pastatest_Type2_breakspasta_sum(ACTIVE,MODEIMPU)
Pastatest_Type2_dropflip_sum(ACTIVE,MODEIMPU)
WP_Visible_Latency_AVG(ACTIVE,MODEIMPU)
WP_Visible_Distance_AVG(ACTIVE,MODEIMPU)
TM_Visible_Latency_AVG(ACTIVE,MODEIMPU)
TM_Visible_Distance_AVG(ACTIVE,MODEIMPU)
WPTM_Visible_Latency_AVG(ACTIVE,MODEIMPU)
WPTM_Visible_Distance_AVG(ACTIVE,MODEIMPU)
WP_Hidden_Latency_Day_AVG(ACTIVE,MODEIMPU)
WP_Hidden_Distance_Day_AVG(ACTIVE,MODEIMPU)

```
TM_Hidden_Latency_Day_AVG(ACTIVE,MODEIMPU)
TM_Hidden_Distance_Day_AVG(ACTIVE,MODEIMPU)
WPTM_Hidden_Latency_Day_AVG(ACTIVE,MODEIMPU)
WPTM_Hidden_Distance_Day_AVG(ACTIVE,MODEIMPU)
WP_Probe_Distance(ACTIVE,MODEIMPU)
TM_Probe_Latency(ACTIVE,MODEIMPU)
TM_Probe_Distance(ACTIVE,MODEIMPU)
WPTM_Probe_Distance(ACTIVE,MODEIMPU)
LP_Probe_Frequency(ACTIVE,MODEIMPU)
LP_Probe_Latency_FirstOcc(ACTIVE,MODEIMPU)
P_Probe_Frequency(ACTIVE,MODEIMPU)
P_Probe_Latency_FirstOcc(ACTIVE,MODEIMPU)
TQ_Probe_Duration(ACTIVE,MODEIMPU)
TQ_Probe_Latency_FirstOcc(ACTIVE,MODEIMPU)
TQ_Probe_PercTime(ACTIVE,MODEIMPU)
  /DIMENSION=3
  /NORMALIZATION=VPRINCIPAL
  /MAXITER=100
  /CRITITER=.00001
/RESAMPLE=BOOTSTRAP (2000 95 BALANCED PROCRU)
  /PRINT DESCRIP VAF LOADING CORR OCORR OBJECT
  /PLOT=BIPLOT(LOADING) (20) OBJECT (20)
/SAVE=OBJECT.
```

SPSS Syntax for 6-dimensional NL-PCA: Double-combo Mino, Double-combo LM11A-31 and Triple-combo experiments (not shown)

```
CATPCA VARIABLES=
Weight_Day_0
Weight_Day_10
BrainLesion_percentage
SLT_Time2Remove_Leftpaw_Avg
SLT_Time2Remove_Rightpaw_Avg
Cylinder_Leftpaw_perc
Cylinder_Rightpaw_perc
Pastatest_Type1_Total_sum
Pastatest_Type1_handsapart_sum
Pastatest_Type1_hunching_sum
Pastatest_Type1_headtilts_sum
Pastatest_Type2_Total_sum
Pastatest_Type2_Switchhands_sum
Pastatest_Type2_usesmouth_sum
Pastatest_Type2_breakspasta_sum
```

Pastatest_Type2_dropflip_sum
WP_Visible_Latency_AVG
WP_Visible_Distance_AVG
TM_Visible_Latency_AVG
TM_Visible_Distance_AVG
WPTM_Visible_Latency_AVG
WPTM_Visible_Distance_AVG
WP_Hidden_Latency_Day_AVG
WP_Hidden_Distance_Day_AVG
TM_Hidden_Latency_Day_AVG
TM_Hidden_Distance_Day_AVG
WPTM_Hidden_Latency_Day_AVG
WPTM_Hidden_Distance_Day_AVG
WP_Probe_Distance
TM_Probe_Latency
TM_Probe_Distance
WPTM_Probe_Distance
LP_Probe_Frequency
LP_Probe_Latency_FirstOcc
P_Probe_Frequency
P_Probe_Latency_FirstOcc
TQ_Probe_Duration
TQ_Probe_Latency_FirstOcc
TQ_Probe_PercTime
/ANALYSIS=
Weight_Day_0(WEIGHT=1,LEVEL= NUME)
Weight_Day_10(WEIGHT=1,LEVEL= NUME)
BrainLesion_percentage(WEIGHT=1,LEVEL= NUME)
SLT_Time2Remove_Leftpaw_Avg(WEIGHT=1,LEVEL= NUME)
SLT_Time2Remove_Rightpaw_Avg(WEIGHT=1,LEVEL= NUME)
Cylinder_Leftpaw_perc(WEIGHT=1,LEVEL= NUME)
Cylinder_Rightpaw_perc(WEIGHT=1,LEVEL= NUME)
Pastatest_Type1_Total_sum(WEIGHT=1,LEVEL= ORDI)
Pastatest_Type1_handsapart_sum(WEIGHT=1,LEVEL= ORDI)
Pastatest_Type1_hunching_sum(WEIGHT=1,LEVEL= ORDI)
Pastatest_Type1_headtilts_sum(WEIGHT=1,LEVEL= ORDI)
Pastatest_Type2_Total_sum(WEIGHT=1,LEVEL= ORDI)
Pastatest_Type2_Switchhands_sum(WEIGHT=1,LEVEL= ORDI)
Pastatest_Type2_usesmouth_sum(WEIGHT=1,LEVEL= ORDI)
Pastatest_Type2_breakspasta_sum(WEIGHT=1,LEVEL= ORDI)
Pastatest_Type2_dropflip_sum(WEIGHT=1,LEVEL= ORDI)
WP_Visible_Latency_AVG(WEIGHT=1,LEVEL= NUME)
WP_Visible_Distance_AVG(WEIGHT=1,LEVEL= NUME)
TM_Visible_Latency_AVG(WEIGHT=1,LEVEL= NUME)

```
TM_Visible_Distance_AVG(WEIGHT=1,LEVEL= NUME)
WPTM_Visible_Latency_AVG(WEIGHT=1,LEVEL= NUME)
WPTM_Visible_Distance_AVG(WEIGHT=1,LEVEL= NUME)
WP_Hidden_Latency_Day_AVG(WEIGHT=1,LEVEL= NUME)
WP_Hidden_Distance_Day_AVG(WEIGHT=1,LEVEL= NUME)
TM_Hidden_Latency_Day_AVG(WEIGHT=1,LEVEL= NUME)
TM_Hidden_Distance_Day_AVG(WEIGHT=1,LEVEL= NUME)
WPTM_Hidden_Latency_Day_AVG(WEIGHT=1,LEVEL= NUME)
WPTM_Hidden_Distance_Day_AVG(WEIGHT=1,LEVEL= NUME)
WP_Probe_Distance(WEIGHT=1,LEVEL= NUME)
TM_Probe_Latency(WEIGHT=1,LEVEL= NUME)
TM_Probe_Distance(WEIGHT=1,LEVEL= NUME)
WPTM_Probe_Distance(WEIGHT=1,LEVEL= NUME)
LP_Probe_Frequency(WEIGHT=1,LEVEL= NUME)
LP_Probe_Latency_FirstOcc(WEIGHT=1,LEVEL= NUME)
P_Probe_Frequency(WEIGHT=1,LEVEL= NUME)
P_Probe_Latency_FirstOcc(WEIGHT=1,LEVEL= NUME)
TQ_Probe_Duration(WEIGHT=1,LEVEL= NUME)
TQ_Probe_Latency_FirstOcc(WEIGHT=1,LEVEL= NUME)
TQ_Probe_PercTime(WEIGHT=1,LEVEL= NUME)
  /DISCRETIZATION=
Weight_Day_0(MULTIPLYING)
Weight_Day_10(MULTIPLYING)
BrainLesion_percentage(MULTIPLYING)
SLT_Time2Remove_Leftpaw_Avg(MULTIPLYING)
SLT_Time2Remove_Rightpaw_Avg(MULTIPLYING)
Cylinder_Leftpaw_perc(MULTIPLYING)
Cylinder_Rightpaw_perc(MULTIPLYING)
Pastatest_Type1_Total_sum(Ranking)
Pastatest_Type1_handsapart_sum(Ranking)
Pastatest_Type1_hunching_sum(Ranking)
Pastatest_Type1_headtilts_sum(Ranking)
Pastatest_Type2_Total_sum(Ranking)
Pastatest_Type2_Switchhands_sum(Ranking)
Pastatest_Type2_usesmouth_sum(Ranking)
Pastatest_Type2_breakspasta_sum(Ranking)
Pastatest_Type2_dropflip_sum(Ranking)
WP_Visible_Latency_AVG(MULTIPLYING)
WP_Visible_Distance_AVG(MULTIPLYING)
TM_Visible_Latency_AVG(MULTIPLYING)
TM_Visible_Distance_AVG(MULTIPLYING)
WPTM_Visible_Latency_AVG(MULTIPLYING)
WPTM_Visible_Distance_AVG(MULTIPLYING)
WP_Hidden_Latency_Day_AVG(MULTIPLYING)
```

WP_Hidden_Distance_Day_AVG(MULTIPLYING)
TM_Hidden_Latency_Day_AVG(MULTIPLYING)
TM_Hidden_Distance_Day_AVG(MULTIPLYING)
WPTM_Hidden_Latency_Day_AVG(MULTIPLYING)
WPTM_Hidden_Distance_Day_AVG(MULTIPLYING)
WP_Probe_Distance(MULTIPLYING)
TM_Probe_Latency(MULTIPLYING)
TM_Probe_Distance(MULTIPLYING)
WPTM_Probe_Distance(MULTIPLYING)
LP_Probe_Frequency(MULTIPLYING)
LP_Probe_Latency_FirstOcc(MULTIPLYING)
P_Probe_Frequency(MULTIPLYING)
P_Probe_Latency_FirstOcc(MULTIPLYING)
TQ_Probe_Duration(MULTIPLYING)
TQ_Probe_Latency_FirstOcc(MULTIPLYING)
TQ_Probe_PercTime(MULTIPLYING)
/MISSING=
Weight_Day_0(ACTIVE,MODEIMPU)
Weight_Day_10(ACTIVE,MODEIMPU)
BrainLesion_percentage(ACTIVE,MODEIMPU)
SLT_Time2Remove_Leftpaw_Avg(ACTIVE,MODEIMPU)
SLT_Time2Remove_Rightpaw_Avg(ACTIVE,MODEIMPU)
Cylinder_Leftpaw_perc(ACTIVE,MODEIMPU)
Cylinder_Rightpaw_perc(ACTIVE,MODEIMPU)
Pastatest_Type1_Total_sum(ACTIVE,MODEIMPU)
Pastatest_Type1_handsapart_sum(ACTIVE,MODEIMPU)
Pastatest_Type1_hunching_sum(ACTIVE,MODEIMPU)
Pastatest_Type1_headtilts_sum(ACTIVE,MODEIMPU)
Pastatest_Type2_Total_sum(ACTIVE,MODEIMPU)
Pastatest_Type2_Switchhands_sum(ACTIVE,MODEIMPU)
Pastatest_Type2_usesmouth_sum(ACTIVE,MODEIMPU)
Pastatest_Type2_breakspasta_sum(ACTIVE,MODEIMPU)
Pastatest_Type2_dropflip_sum(ACTIVE,MODEIMPU)
WP_Visible_Latency_AVG(ACTIVE,MODEIMPU)
WP_Visible_Distance_AVG(ACTIVE,MODEIMPU)
TM_Visible_Latency_AVG(ACTIVE,MODEIMPU)
TM_Visible_Distance_AVG(ACTIVE,MODEIMPU)
WPTM_Visible_Latency_AVG(ACTIVE,MODEIMPU)
WPTM_Visible_Distance_AVG(ACTIVE,MODEIMPU)
WP_Hidden_Latency_Day_AVG(ACTIVE,MODEIMPU)
WP_Hidden_Distance_Day_AVG(ACTIVE,MODEIMPU)
TM_Hidden_Latency_Day_AVG(ACTIVE,MODEIMPU)
TM_Hidden_Distance_Day_AVG(ACTIVE,MODEIMPU)
WPTM_Hidden_Latency_Day_AVG(ACTIVE,MODEIMPU)

```
WPTM_Hidden_Distance_Day_AVG(ACTIVE,MODEIMPU)
WP_Probe_Distance(ACTIVE,MODEIMPU)
TM_Probe_Latency(ACTIVE,MODEIMPU)
TM_Probe_Distance(ACTIVE,MODEIMPU)
WPTM_Probe_Distance(ACTIVE,MODEIMPU)
LP_Probe_Frequency(ACTIVE,MODEIMPU)
LP_Probe_Latency_FirstOcc(ACTIVE,MODEIMPU)
P_Probe_Frequency(ACTIVE,MODEIMPU)
P_Probe_Latency_FirstOcc(ACTIVE,MODEIMPU)
TQ_Probe_Duration(ACTIVE,MODEIMPU)
TQ_Probe_Latency_FirstOcc(ACTIVE,MODEIMPU)
TQ_Probe_PercTime(ACTIVE,MODEIMPU)
  /DIMENSION=6
  /NORMALIZATION=VPRINCIPAL
  /MAXITER=100
  /CRITITER=.00001
/RESAMPLE=BOOTSTRAP (2000 95 BALANCED PROCRU)
  /PRINT DESCRIP VAF LOADING CORR OCORR OBJECT
  /PLOT=BIPLOT(LOADING) (20) OBJECT (20)
/SAVE=OBJECT.
```

SPSS Syntax for 2-dimensional NL-PCA: Double-combo Mino, Double-combo LM11A-31 and Triple-combo experiments (Supplementary Fig. 5a)

```
CATPCA VARIABLES=
Weight_Day_0
Weight_Day_10
BrainLesion_percentage
SLT_Time2Remove_Leftpaw_Avg
SLT_Time2Remove_Rightpaw_Avg
Cylinder_Leftpaw_perc
Cylinder_Rightpaw_perc
Pastatest_Type1_Total_sum
Pastatest_Type1_handsapart_sum
Pastatest_Type1_hunching_sum
Pastatest_Type1_headtilts_sum
Pastatest_Type2_Total_sum
Pastatest_Type2_Switchhands_sum
Pastatest_Type2_usesmouth_sum
Pastatest_Type2_breakspasta_sum
Pastatest_Type2_dropflip_sum
WP_Visible_Latency_AVG
WP_Visible_Distance_AVG
```

```
TM_Visible_Latency_AVG
TM_Visible_Distance_AVG
WPTM_Visible_Latency_AVG
WPTM_Visible_Distance_AVG
WP_Hidden_Latency_Day_AVG
WP_Hidden_Distance_Day_AVG
TM_Hidden_Latency_Day_AVG
TM_Hidden_Distance_Day_AVG
WPTM_Hidden_Latency_Day_AVG
WPTM_Hidden_Distance_Day_AVG
WP_Probe_Distance
TM_Probe_Latency
TM_Probe_Distance
WPTM_Probe_Distance
LP_Probe_Frequency
LP_Probe_Latency_FirstOcc
P_Probe_Frequency
P_Probe_Latency_FirstOcc
TQ_Probe_Duration
TQ_Probe_Latency_FirstOcc
TQ_Probe_PercTime
 /ANALYSIS=
Weight_Day_0(WEIGHT=1,LEVEL= NUME)
Weight_Day_10(WEIGHT=1,LEVEL= NUME)
BrainLesion_percentage(WEIGHT=1,LEVEL= NUME)
SLT_Time2Remove_Leftpaw_Avg(WEIGHT=1,LEVEL= NUME)
SLT_Time2Remove_Rightpaw_Avg(WEIGHT=1,LEVEL= NUME)
Cylinder_Leftpaw_perc(WEIGHT=1,LEVEL= NUME)
Cylinder_Rightpaw_perc(WEIGHT=1,LEVEL= NUME)
Pastatest_Type1_Total_sum(WEIGHT=1,LEVEL= ORDI)
Pastatest_Type1_handsapart_sum(WEIGHT=1,LEVEL= ORDI)
Pastatest_Type1_hunching_sum(WEIGHT=1,LEVEL= ORDI)
Pastatest_Type1_headtilts_sum(WEIGHT=1,LEVEL= ORDI)
Pastatest_Type2_Total_sum(WEIGHT=1,LEVEL= ORDI)
Pastatest_Type2_Switchhands_sum(WEIGHT=1,LEVEL= ORDI)
Pastatest_Type2_usesmouth_sum(WEIGHT=1,LEVEL= ORDI)
Pastatest_Type2_breakspasta_sum(WEIGHT=1,LEVEL= ORDI)
Pastatest_Type2_dropflip_sum(WEIGHT=1,LEVEL= ORDI)
WP_Visible_Latency_AVG(WEIGHT=1,LEVEL= NUME)
WP_Visible_Distance_AVG(WEIGHT=1,LEVEL= NUME)
TM_Visible_Latency_AVG(WEIGHT=1,LEVEL= NUME)
TM_Visible_Distance_AVG(WEIGHT=1,LEVEL= NUME)
WPTM_Visible_Latency_AVG(WEIGHT=1,LEVEL= NUME)
WPTM_Visible_Distance_AVG(WEIGHT=1,LEVEL= NUME)
```

```
WP_Hidden_Latency_Day_AVG(WEIGHT=1,LEVEL= NUME)
WP_Hidden_Distance_Day_AVG(WEIGHT=1,LEVEL= NUME)
TM_Hidden_Latency_Day_AVG(WEIGHT=1,LEVEL= NUME)
TM_Hidden_Distance_Day_AVG(WEIGHT=1,LEVEL= NUME)
WPTM_Hidden_Latency_Day_AVG(WEIGHT=1,LEVEL= NUME)
WPTM_Hidden_Distance_Day_AVG(WEIGHT=1,LEVEL= NUME)
WP_Probe_Distance(WEIGHT=1,LEVEL= NUME)
TM_Probe_Latency(WEIGHT=1,LEVEL= NUME)
TM_Probe_Distance(WEIGHT=1,LEVEL= NUME)
WPTM_Probe_Distance(WEIGHT=1,LEVEL= NUME)
LP_Probe_Frequency(WEIGHT=1,LEVEL= NUME)
LP_Probe_Latency_FirstOcc(WEIGHT=1,LEVEL= NUME)
P_Probe_Frequency(WEIGHT=1,LEVEL= NUME)
P_Probe_Latency_FirstOcc(WEIGHT=1,LEVEL= NUME)
TQ_Probe_Duration(WEIGHT=1,LEVEL= NUME)
TQ_Probe_Latency_FirstOcc(WEIGHT=1,LEVEL= NUME)
TQ_Probe_PercTime(WEIGHT=1,LEVEL= NUME)
  /DISCRETIZATION=
Weight_Day_0(MULTIPLYING)
Weight_Day_10(MULTIPLYING)
BrainLesion_percentage(MULTIPLYING)
SLT_Time2Remove_Leftpaw_Avg(MULTIPLYING)
SLT_Time2Remove_Rightpaw_Avg(MULTIPLYING)
Cylinder_Leftpaw_perc(MULTIPLYING)
Cylinder_Rightpaw_perc(MULTIPLYING)
Pastatest_Type1_Total_sum(Ranking)
Pastatest_Type1_handsapart_sum(Ranking)
Pastatest_Type1_hunching_sum(Ranking)
Pastatest_Type1_headtilts_sum(Ranking)
Pastatest_Type2_Total_sum(Ranking)
Pastatest_Type2_Switchhands_sum(Ranking)
Pastatest_Type2_usesmouth_sum(Ranking)
Pastatest_Type2_breakspasta_sum(Ranking)
Pastatest_Type2_dropflip_sum(Ranking)
WP_Visible_Latency_AVG(MULTIPLYING)
WP_Visible_Distance_AVG(MULTIPLYING)
TM_Visible_Latency_AVG(MULTIPLYING)
TM_Visible_Distance_AVG(MULTIPLYING)
WPTM_Visible_Latency_AVG(MULTIPLYING)
WPTM_Visible_Distance_AVG(MULTIPLYING)
WP_Hidden_Latency_Day_AVG(MULTIPLYING)
WP_Hidden_Distance_Day_AVG(MULTIPLYING)
TM_Hidden_Latency_Day_AVG(MULTIPLYING)
TM_Hidden_Distance_Day_AVG(MULTIPLYING)
```

```
WPTM_Hidden_Latency_Day_AVG(MULTIPLYING)
WPTM_Hidden_Distance_Day_AVG(MULTIPLYING)
WP_Probe_Distance(MULTIPLYING)
TM_Probe_Latency(MULTIPLYING)
TM_Probe_Distance(MULTIPLYING)
WPTM_Probe_Distance(MULTIPLYING)
LP_Probe_Frequency(MULTIPLYING)
LP_Probe_Latency_FirstOcc(MULTIPLYING)
P_Probe_Frequency(MULTIPLYING)
P_Probe_Latency_FirstOcc(MULTIPLYING)
TQ_Probe_Duration(MULTIPLYING)
TQ_Probe_Latency_FirstOcc(MULTIPLYING)
TQ_Probe_PercTime(MULTIPLYING)
/MISSING=
Weight_Day_0(ACTIVE,MODEIMPU)
Weight_Day_10(ACTIVE,MODEIMPU)
BrainLesion_percentage(ACTIVE,MODEIMPU)
SLT_Time2Remove_Leftpaw_Avg(ACTIVE,MODEIMPU)
SLT_Time2Remove_Rightpaw_Avg(ACTIVE,MODEIMPU)
Cylinder_Leftpaw_perc(ACTIVE,MODEIMPU)
Cylinder_Rightpaw_perc(ACTIVE,MODEIMPU)
Pastatest_Type1_Total_sum(ACTIVE,MODEIMPU)
Pastatest_Type1_handsapart_sum(ACTIVE,MODEIMPU)
Pastatest_Type1_hunching_sum(ACTIVE,MODEIMPU)
Pastatest_Type1_headtilts_sum(ACTIVE,MODEIMPU)
Pastatest_Type2_Total_sum(ACTIVE,MODEIMPU)
Pastatest_Type2_Switchhands_sum(ACTIVE,MODEIMPU)
Pastatest_Type2_usesmouth_sum(ACTIVE,MODEIMPU)
Pastatest_Type2_breakspasta_sum(ACTIVE,MODEIMPU)
Pastatest_Type2_dropflip_sum(ACTIVE,MODEIMPU)
WP_Visible_Latency_AVG(ACTIVE,MODEIMPU)
WP_Visible_Distance_AVG(ACTIVE,MODEIMPU)
TM_Visible_Latency_AVG(ACTIVE,MODEIMPU)
TM_Visible_Distance_AVG(ACTIVE,MODEIMPU)
WPTM_Visible_Latency_AVG(ACTIVE,MODEIMPU)
WPTM_Visible_Distance_AVG(ACTIVE,MODEIMPU)
WP_Hidden_Latency_Day_AVG(ACTIVE,MODEIMPU)
WP_Hidden_Distance_Day_AVG(ACTIVE,MODEIMPU)
TM_Hidden_Latency_Day_AVG(ACTIVE,MODEIMPU)
TM_Hidden_Distance_Day_AVG(ACTIVE,MODEIMPU)
WPTM_Hidden_Latency_Day_AVG(ACTIVE,MODEIMPU)
WPTM_Hidden_Distance_Day_AVG(ACTIVE,MODEIMPU)
WP_Probe_Distance(ACTIVE,MODEIMPU)
TM_Probe_Latency(ACTIVE,MODEIMPU)
```

```
TM_Probe_Distance(ACTIVE,MODEIMPU)
WPTM_Probe_Distance(ACTIVE,MODEIMPU)
LP_Probe_Frequency(ACTIVE,MODEIMPU)
LP_Probe_Latency_FirstOcc(ACTIVE,MODEIMPU)
P_Probe_Frequency(ACTIVE,MODEIMPU)
P_Probe_Latency_FirstOcc(ACTIVE,MODEIMPU)
TQ_Probe_Duration(ACTIVE,MODEIMPU)
TQ_Probe_Latency_FirstOcc(ACTIVE,MODEIMPU)
TQ_Probe_PercTime(ACTIVE,MODEIMPU)
  /DIMENSION=2
  /NORMALIZATION=VPRINCIPAL
  /MAXITER=100
  /CRITITER=.00001
/RESAMPLE=BOOTSTRAP (2000 95 BALANCED PROCRU)
  /PRINT DESCRIP VAF LOADING CORR OCORR OBJECT
  /PLOT=BIPLOT(LOADING) (20) OBJECT (20)
/SAVE=OBJECT.
```
